# Dual Role of Integrin Alpha-6 in Glioblastoma: Supporting Stemness in Proneural Stem-Like Cells While Inducing Radioresistance in Mesenchymal Stem-Like Cells

**DOI:** 10.3390/cancers13123055

**Published:** 2021-06-19

**Authors:** Elisabetta Stanzani, Leire Pedrosa, Guillaume Bourmeau, Oceane Anezo, Aleix Noguera-Castells, Anna Esteve-Codina, Lorena Passoni, Michela Matteoli, Núria de la Iglesia, Giorgio Seano, Fina Martínez-Soler, Avelina Tortosa

**Affiliations:** 1 Apoptosis and Cancer Unit, Department of Physiological Sciences, IDIBELL, Faculty of Medicine and Health Sciences, Universitat de Barcelona, 08907 L’Hospitalet del Llobregat, Spain; finamartinez@ub.edu; 2 Haematology and Oncology Unit, August Pi i Sunyer Biomedical Research Institute (IDIBAPS), 08036 Barcelona, Spain; lepedrosa@clinic.cat (L.P.); ndelaiglesia@irsicaixa.es (N.d.l.I.); 3 Tumor Microenvironment Lab., Institut Curie, Université PSL, Université Paris-Saclay, CNRS UMR3347, Inserm U1021, Signalisation Radiobiologie et Cancer, 91400 Orsay, France; guillaume.bourmeau@curie.fr (G.B.); oceane.carriere@curie.fr (O.A.); giorgio.seano@curie.fr (G.S.); 4 Laboratory of Molecular and Translational Oncology, Departament of Medicine, CELLEX Biomedical Research Centre, Faculty of Medicine and Health Sciences, Universitat de Barcelona, 08036 Barcelona, Spain; anogueca29@alumnes.ub.edu; 5 Functional Genomics, Centre for Genomic Regulation (CNAG-CRG), Barcelona Institute of Science and Technology, 08028 Barcelona, Spain; anna.esteve@cnag.crg.eu; 6 Universitat Pompeu Fabra (UPF), 08003 Barcelona, Spain; 7 Laboratory of Pharmacology and Brain Pathology, IRCCS Humanitas Research Hospital, 20089 Rozzano, Italy; lorena.passoni@humanitasresearch.it; 8 CNR Institute of Neuroscience, c/o Humanitas, 20089 Rozzano, Italy; michela.matteoli@hunimed.eu; 9 Department of Basic Nursing, Faculty of Medicine and Health Sciences, Universitat de Barcelona, 08907 L’Hospitalet del Llobregat, Spain

**Keywords:** glioblastoma, integrin alpha-6, *ITGA6*, radiotherapy, cancer stem cells, mesenchymal subtype, radioresistance

## Abstract

**Simple Summary:**

Glioblastoma stem-like cells (GSCs) are responsible for most of the malignant characteristics of glioblastoma, including therapeutic resistance, tumour recurrence, and tumour cellular heterogeneity. Therefore, increased understanding of the mechanisms regulating GSCs aggressiveness may help to improve patients’ outcomes. Here, we investigated the role of integrin a6 in controlling stemness and resistance to radiotherapy across proneural and mesenchymal molecular subtypes. We observed that integrin a6 had a clear role in stemness maintenance in proneural but not in mesenchymal GSCs. In addition, we proved a crucial role of integrin a6 in supporting mesenchymal GSCs resistance to ionizing radiation. Finally, we highlighted that integrin a6 may control different stem-associated features in GSCs, depending on the molecular subtype. The inhibition of integrin a6 limits stem-like malignant characteristics in both GSCs subtypes and thus may potentially control tumour relapse following conventional treatment.

**Abstract:**

Therapeutic resistance after multimodal therapy is the most relevant cause of glioblastoma (GBM) recurrence. Extensive cellular heterogeneity, mainly driven by the presence of GBM stem-like cells (GSCs), strongly correlates with patients’ prognosis and limited response to therapies. Defining the mechanisms that drive stemness and control responsiveness to therapy in a GSC-specific manner is therefore essential. Here we investigated the role of integrin a6 (*ITGA6*) in controlling stemness and resistance to radiotherapy in proneural and mesenchymal GSCs subtypes. Using cell sorting, gene silencing, RNA-Seq, and in vitro assays, we verified that *ITGA6* expression seems crucial for proliferation and stemness of proneural GSCs, while it appears not to be relevant in mesenchymal GSCs under basal conditions. However, when challenged with a fractionated protocol of radiation therapy, comparable to that used in the clinical setting, mesenchymal GSCs were dependent on integrin a6 for survival. Specifically, GSCs with reduced levels of *ITGA6* displayed a clear reduction of DNA damage response and perturbation of cell cycle pathways. These data indicate that *ITGA6* inhibition is able to overcome the radioresistance of mesenchymal GSCs, while it reduces proliferation and stemness in proneural GSCs. Therefore, integrin a6 controls crucial characteristics across GBM subtypes in GBM heterogeneous biology and thus may represent a promising target to improve patient outcomes.

## 1. Introduction

Glioblastoma (GBM) is the most common and most malignant primary brain tumour in adults. It is characterized by high recurrence rates even after maximal resection and multimodal treatment [1]. Despite many efforts to delineate new therapeutical strategies, radiotherapy remains the most successful non-surgical treatment for GBM associated with the best survival benefit [2,3]. Conventional radiation protocol involves fractionated focal radiotherapy (maximum dose 60 Gy, 1.8–2.0 Gy/fraction per day) [1].

From the histological point of view, GBMs display neoplastic lesions with remarkable cellular heterogeneity [4]. Key players among the various cellular elements are the GBM stem-like cells (GSCs), which are commonly believed to be at the origin of tumorigenesis, invasion, angiogenesis, immune evasion, and treatment resistance [5,6,7,8,9,10,11]. Notably, GSCs are characterized by specific biological features which confer them an outstanding capacity to cope with radiation-induced cell-damage [7,12,13].

Generally, GSCs are located in trophic niches [6,14,15] and share most of the core characteristics with non-transformed stem cells, including self-renewal capability, extensive proliferation and multipotency [5,11,16]. Among the mediators granting the interaction between GSCs and the surrounding microenvironment, integrins are important elements. Integrins are cell type-I transmembrane heterodimers composed of different combinations of alpha (a) and beta (b) subunits [17]. Integrins shape the niche architecture and are indeed involved in stem cell proliferation and self-renewal, homing, and maintenance in the niche [18].

The integrin subunit a6 (coded by the gene *ITGA6*) has received considerable attention, especially for its role in regulating cancer stem-like cells [19,20]. Integrin a6 dimerizes with integrin b1 or b4 to generate the surface receptor for laminin. In human mesenchymal stem cells, integrin a6 has been described as maintaining pluripotency through prolonged activation of the PI3K/AKT pathway and sustained expression of *OCT4* and *SOX2* [21]. In GBM, *ITGA6* is commonly used as a GSCs marker, being capable of enriching for the GSCs population, alone or in combination with CD133, and also sustaining stemness [22]. More recently, integrin a6 has been associated with *ZEB1* transcriptional network to sustain DNA damage response in GBM [23]. 

In the last decade, GBM patient specimens have been inspected to identify gene expression profiles that could allow patient stratification and therapy response prediction. Following a progressive optimization, three molecular subgroups were consistently identified: mesenchymal (MES), proneural (PN), and classical (CL) [24,25]. GSCs harboured within tumour samples reflect similar transcriptional clusters, with PN and MES being the most consolidated profiles [26,27,28,29]. Although the PN subtype tends to be associated with a more favourable outcome, the molecular patterns of GBM only partially explain clinical behaviour and their predictive power is scarce [30]. 

To our knowledge, the role of integrin a6 in the GBM context has never been investigated in relation to GBM molecular heterogeneity. In breast cancer, integrin a6 expression has been related to a distinct role in tumoral cells bearing epithelial or mesenchymal phenotype [31]. Indeed, integrin a6 has been mostly characterized in the GBM context in association with common proneural GSCs markers, such as transcription factor SOX2 and Oligo2 [23] or CD133 and Oligo2 [22]. However, there is still no indication on the role of this membrane receptor in MES-GSCs. 

Here we investigated the implications of *ITGA6* expression in GSCs biology according to the different transcriptional subtypes PN and MES. Given the extensive body of evidence on PN-GSCs, we focused on assessing the role of integrin a6 in MES-GSCs. We observed that integrin a6 supported stemness in PN but not in MES settings. *ITGA6* downregulation affected DNA damage repair machinery and cell cycle in the MES profile, thus reducing the capacity to clear gamma-H2AX foci upon ionizing radiation and, therefore, increasing radiosensitivity. 

## 2. Materials and Methods

### 2.1. Human Cell Lines and Differentiation Assay

Glioblastoma stem-like cells (GSCs) cultures were isolated from post-surgical specimens from consenting GBM patients (histological diagnosis GBM WHO grade IV, IDH1-wt). Collection of human samples was performed according to the protocol approved by the Ethics Committee of Hospital Universitari de Bellvitge. Tumoral samples were processed as previously described [32]. GSCs cultures were established following the neurosphere culture method without selection of specific stem markers [33,34]. Briefly, tissues were enzymatically dissociated for 30 min at 37 °C with 20 U/mL Papain (Worthington Biochemical Corporation, Lakewood, NJ, USA), stabilized with 8.25 µM l-cysteine (Sigma-Aldrich, St. Louis, MO, USA) and 3.42 µM EDTA (Panreac Química S.L.U., Castellar del Vallés, Spain). The cell suspension was cultured and maintained in FBS-free media supplemented with EGF 20 ng/mL (PeproTech EC, Ltd., London, UK) and bFGF 10 ng/mL (PrepoTech). Out of seven GBM patient specimens, four different MES-GSC cultures were successfully established (57% efficiency of isolation). MES-GSC cultures were differentiated in 10% FBS media, and after 7 days, samples were collected. Differentiated glioblastoma cells (DGCs) were established from the same human post-surgical specimens of GSCs, as described before [32], and cultured in 10% FBS DMEM (Biological Industries, Kibbutz Beit-Haemek, Israel). All experiments were performed before passage 20 and tested to be mycoplasma negative. HEK293T were maintained following the ATCC guidelines. PN-GIC2 and PN-GIC7 were kindly provided by Marta Alonso (Department of Oncology, University Hospital of Navarra, Pamplona, Spain) and Candelaria Gómez-Manzano (Department of Neuro-Oncology, The University of Texas, MD Anderson Cancer Center, Houston, TX, USA).

### 2.2. RNA Isolation and Real-Time q-PCR Analyses

Total RNA was extracted from samples using TRIsure (Bioline) and subsequently digested with DNase I RNase-free (Thermo Scientific, Waltham, MA, USA). RNA purity and concentration were measured using Nanodrop spectrophotometer (Thermo Scientific). Reverse-transcription of RNA was performed using the High-Capacity cDNA Reverse Transcription Kit (Life Technologies, Carlsbad, CA, USA). Gene expression was determined using validated Taqman^®^ Gene Expression Assays (Applied Biosystems, Waltham, MA, USA; GAPDH: Hs99999905_m1; GUSB: Hs00939627_m1; CD44: Hs1075864_m1; OLIG2: Hs00300164_s1; PROM1: Hs01009257_m1; ITGA6: Hs01041011_m1; NES: Hs04187831_g1; ALDH1A3: Hs00167476_m1) and 7900HT Real-Time RT-PCR system (Applied Biosystems). Cycling conditions were as default for Taqman^®^ probes: 50 °C for 2 min, 95 °C for 10 min, followed by 40 cycles of 15 s at 95 °C for denaturation and 1 min at 60 °C for annealing and extension. Relative mRNA content was calculated based on the ddCt method, while the normalized mRNA amount was obtained with the dCt process. Normalization in both approaches was performed with *GAPDH* and *GUSB* as housekeeping genes.

### 2.3. Flow Cytometry and Integrin a6 Cell Sorting

GSCs were collected, rinsed and blocked with FACS buffer (0.5% BSA, 2 mM EDTA dissolved in PBS; pH 7.2) for 15 min at room temperature. Cells were stained with unconjugated anti-integrin a6 antibodies (1/100; clone NKI-GoH3; Millipore, Burlington, MA, USA) for 15 min at room temperature. Bound antibodies were then revealed by species-specific Alexa Fluor conjugated secondary antibodies (Invitrogen, Waltham, MA, USA). As DGCs cultures do not express integrin a6 [32], they were used as negative controls. The stained cells were acquired on FACS Canto (BD Biosciences, Franklin Lakes, NJ, USA) using FACS Diva software (Becton Dickinson, Franklin Lakes, NJ, USA). Data analysis and median fluorescence intensity (MFI) calculation were performed using the FlowJo software (Tree Star Inc. Ashland, OR, USA). ITGA6HI and ITGA6LO cell populations were obtained using MoFlo Astrios cell sorter (Beckman Coulter, Brea, CA, USA) on the basis of ITGA6-MFI cells.

### 2.4. Western Blot

Proteins were extracted in reducing condition with 0.3% CHAPs (Sigma-Aldrich) lysis buffer supplemented with protease inhibitors (Complete and PhosSTOP from Roche). Antibodies against b-actin (1/5000; clone AC-15; Sigma-Aldrich), integrin a6 (1/500; clone HPA012696; Novus Biologicals, Littleton, CO, USA), and p-ERK1/2 (1/2000; clone #4370; Cell Signalling Technology, Danvers, MA, USA) were used. Densitometric analysis was carried out using Multi Gauge software (FujiFilm Corporation, Tokyo, Japan).

### 2.5. Gliomasphere Formation Assay

To assess GSCs self-renewal capacity, cells were seeded in 96-well plates as single-cell suspension at clonal densities 0.2–0.4 cell/µL in quadruplicate. Cells were allowed to proliferate for 14 days, and then plates were visually scanned under light microscope, and the gliomasphere size was recorded. Gliomaspheres bigger than 90 μm were scored as proliferating (ProgRes CapturePro, Jenoptik, Huntsville, AL, USA). 

### 2.6. Lentiviral Particle Generation and GSCs Transduction

To silence the integrin a6 expression, GSCs were treated with short hairpin (sh)RNA delivered via lentiviral vectors. Non-targeting shRNA construct (shCTRL; source clone ID: RHS4348) and shITGA6 plasmid mapping against *ITGA6* exon 15 (target sequence: AGGATATTGCTTTAGAAAT; source clone ID: V3LHS_326014) were purchased from Thermo-Scientific. The plasmids used display target insert under the hCMV promoter cloned into the pGIPZ backbone. Lentiviral particles were generated by co-transfection of the plasmids of interest with viral packaging vectors (VSVG, RSV-REV, and pMDL g/p RRE) into HEK293T cells using the GenJet Plus DNA Transfection Reagent (SignaGen Laboratories, Rockville, MD, USA). Cell culture supernatant, once collected, was filtered (0.45 µm) and purified by centrifugation at 40,000× *g* for 2 h at 4 °C. Particles were resuspended in PBS and GSCs infected for 4 h at 37 °C. To ease the transduction, Polybrene was added at 5 μg/mL (Hexadimethrine bromide, Sigma-Aldrich). Infected cells were selected with 5 µg/mL of puromycin (Sigma-Aldrich). Efficient silencing of *ITGA6* transcript was assessed by PCR analysis at all times.

### 2.7. Radiation Schedule

In vitro irradiation of GSCs was achieved using an X-ray accelerator (Clinac 600 CD, M/S Varian AG) at a dose-rate of 2.67 Gy/min. Dosimetry calculations were performed by the Medical Physics Department at the Catalan Institute of Oncology. For the evaluation of GSCs DNA repair capacity (gamma-H2AX foci formation assay), GSCs were irradiated with 4.0 Gy delivered in single dose. To evaluate the capacity of GSCs to functionally recover from irradiation and form gliomaspheres (clonogenic assay and ELDA), GSCs were irradiated following a fractionated protocol (2.0 Gy every 24 h). Total absorbed dose was 2.0 or 4.0 Gy for the clonogenic assay and 8.0 Gy for ELDA.

### 2.8. Gamma-H2AX Foci Formation Assay

GSCs were seeded on glass coverslips and allowed to adhere 24 h prior to treatment. At the indicated times following RT (1, 4, and 24 h), the cells were fixed in 4% paraformaldehyde and permeabilized with 0.1% Triton-X/PBS. Cells were subsequently stained with anti-gamma-H2AX antibody (Ser139; 1/400; Millipore; JBW301 Billerica, MA, USA). To visualize gamma-H2AX foci, samples were incubated with Cy3-conjugated secondary antibody (1/500; Jackson ImmunoResearch, West Grove, PA, USA). Nuclei were counterstained with DRAQ5 (1/2500; Biostatus, Leicestershire, UK) Micrographs were acquired with a Leica TCS-SL Spectral confocal microscope (Leica Microsystems, Wetzlar, Germany) ). Nuclei and foci were counted using Image J (U.S. National Institutes of Health, https://imagej.nih.gov/ij/, accessed on 25 July 2017). An average of five to eight micrographs were analysed for each treatment condition.

### 2.9. Clonogenic Assay and Linear-Quadratic Model Interpretation

For survival quantitation following RT, GSCs were analysed as previously described [32]. Briefly, cells were seeded at clonal density 0.2–0.4 cell/µL in quadruplicate and then exposed to an RT schedule. GSCs were treated following a fractionated protocol. Cells were cultured for 14 days and the total number of newly formed gliomaspheres bigger than 90 μm was recorded. The survival curves obtained were compared according to the published extensive guidelines [35]. Briefly, the surviving fraction (SF) at each treatment dose (D) was calculated (SF2 at 2.0 Gy and SF4 at 4.0 Gy) as the ratio of gliomaspheres arising in treated samples in comparison with control untreated cells. SF were then fit according to the linear–quadratic model (LQM): SF = exp – (αD + βD^2^) [35,36]. Differential radiosensitivity was evaluated via comparison of SF2, SF4, the area under the curve (AUC), and the LQM parameters: α- and β-values and α/β [32,35,37,38].

### 2.10. In-Vitro Extreme Limiting Dilution Assay (ELDA)

To obtain single cell suspension, GSCs were mechanically dissociated and filtered with a 40 µm cell strainer (BD Biosciences). Cells were seeded into a 96-well plate at 1, 5, 10, 20 cells per well, with at least 10 replicates for each condition. The exact number of cells seeded was determined after 24 h in a bright field inverted microscope. Cells were then irradiated following a fractionated schedule. After 14 days the number of gliomaspheres/well (size cut-off: 90 µm) was scored. Stem cell frequency in each samples was calculated using the Extreme Limiting Dilution Analysis web tool (http://bioinf.wehi.edu.au/software/elda, accessed on 12 July 2017) [39].

### 2.11. Bulk RNA-Seq and Bioinformatic Analysis

Total RNA was extracted from samples and quantity/quality was assessed using an Agilent LabChip instrument showing excellent integrity (RNA Integration Number, RIN > 9). purity based on absorbance ratios (260/280 and 260/230) was checked using a Nanodrop spectrophotometer. Then, 700 ng of total RNA was used to prepare RNA sequencing libraries via Illumina TruSeq Stranded mRNA Library preparation kit, which allows creation of libraries for strand-specific mRNA sequencing. 

First, to address sequencing specifically on polyadenylated transcripts, magnetic beads were used for a polyA selection. Next, after fragmentation, cDNA was synthesized, dA-tailed and ligated to TruSeq indexed adapters (unique dual indexing strategy). Finally, PCR amplification was used to create the final cDNA library. After qPCR quantification, sequencing was carried out using 2 × 100 cycles (paired-end reads of 100 nucleotides each) on an Illumina NovaSeq 6000 system (S1 flow cells). Around 50 M paired-end reads were obtained per sample. 

Bcl2fastq was used to generate fastq files from raw sequencing data, where demultiplexing was performed according to indexes.

The Institut Curie RNA-seq bioinformatics pipeline (available at https://github.com/bioinfo-pf-curie/RNA-seq, accessed on 7 March 2021 (v0.2.4)) was used to process sequencing reads. Briefly, potential rRNA contamination was cleaned by sequencing reads on the rRNA human complete reference (U13369.1). Then, by using STAR (v2.5.3a) and the GENCODE annotation database (v19) with the following parameters (—outFilterType BySJout—outFilterMultimapNmax 20—alignSJoverhangMin 8—alignSJDBoverhangMin 1—outFilterMismatchNmax 999—outFilterMismatchNoverLmax 0.04—alignIntronMin 20—alignIntronMax 1,000,000—alignMatesGapMax 1,000,000—outSAMprimaryFlag OneBestScore—outMultimapperOrder Random) the remaining reads were then aligned on the human reference genome (hg19). Finally, the raw gene counts tables were also generated by STAR, using the option –quantMode GeneCounts.

Differential gene expression analysis on raw gene counts was performed using the DESeq2 suite of analysis tools, v1.30.0 [40]. The EnhancedVolcano R package was used to create volcano plots, by means of the fold change and adjusted *p*-values of the genes, obtained using the DESeq2 suite. The fold change of the genes was also used to create graphic representations of pathway enrichment, using the pathview R package. Gene set enrichment analysis (GSEA) was performed using the public Broad Institute software and its publicly available molecular signatures database (KEGG, HALLMARK, Gene Ontology) with raw gene counts data as input. The software computes logFoldChange and *p*-values of genes in a given comparison, ranks them, and scores the genes according to their rank. It gives an enrichment score (ES) depending on the over-representation of genes of a given predefined gene set in one of the conditions. This score is normalized (NES) and a statistical value is given (*p*-value). The RNA-seq data were deposited under the GEO reference GSE178260. Transcription factor enrichment analysis was performed using Enrichr [41]. For analysis of the activated/inhibited pathways, we used Ingenuity Pathway Analysis (IPA, Ingenuity Systems, Inc., Redwood City, CA, USA). Molecules from the data set that were associated with Ingenuity Knowledge Base were considered for the analysis. We included only the significantly differentially expressed genes with no cutoff for the fold change.

### 2.12. Elaboration of Publicly Available Data from GBM Patients

The integrin a6 gene expression pattern across glioma patients was investigated using publicly available cohorts. The Cancer Genome Atlas (TCGA) [42] and REMBRANDT datasets [43] were downloaded from the GlioVis portal (http://gliovis.bioinfo.cnio.es/, accessed on 20 March 2020) [44]. Samples were classified according to molecular subtype following available GlioVis tool as described in [25]. Gene expression data of the GSCs collection were retrieved from Gene Expression Omnibus (GEO). The two independent interrogated datasets were GEO accession GSE49009 (Bhat cohort; 11 PN-GSCs and 6 MES-GSCs) [26] and GSE67089 (Mao cohort; 6 PN-GSCs and 4 MES-GSCs) [27]. For the *ITGA6* expression in the two-dimensional representation according to the new integrative GBM classification model [45], refer to the Broad Institute Single Cell Portal website (https://singlecell.broadinstitute.org/single_cell, accessed on 29 September 2020), study number SCP393 (load cell state hierarchy plot with gene expression annotation). 

Survival analysis and risk assessment of *ITGA6*-associated signature for 660 GBM and low grade gliomas were performed with the SurvExpress web-source (http://bioinformatica.mty.itesm.mx:8080/Biomatec/SurvivaX.jsp, accessed on 10 November 2020) [46].

### 2.13. Statistical Analysis

Data graphs are presented as means ± SEM (standard error of the mean). Statistical analyses were performed using the GraphPad Prism^®^ software. Statistical tests used for each experimental data collection are specified in each figure caption. Calculated *p*-value is summarized in figure panels as * *p* < 0.05; ** *p* < 0.01; *** *p* < 0.001; **** *p* < 0.0001. Different tests were used to assess statistical significance according to data distributions and to the nature of comparison (unpaired *t*-test with or without Welch’s correction, Mann–Whitney test, and two-way ANOVA). Every test used is specified for each panel in the figure caption.

## 3. Results

### 3.1. Integrin a6 Is Expressed in Proneural and Mesenchymal GBM Subtypes

To better understand the significance of integrin a6 in GBM, we analysed in silico the *ITGA6* expression in the two most important GBM subtypes. Data extracted from two distinct datasets of GBM patients [42,43] showed that *ITGA6* expression was greater in GBMs compared with non-cancerous samples (Figure S1A) and that MES subtype expresses significantly higher levels of *ITGA6* than PN GBMs (Figure S1B). In addition, in line with this, *ITGA6* expression was greater in the MES-GSCs in a dataset obtained from in vitro patient-derived GSCs [27] (Figure S1C). Even taking into consideration the recent integrative model that tuned the previous classification into four molecular subtypes [45], *ITGA6*-expressing cells were specifically enriched in the MES-like state (Figure S1D). This independent evidence prompted us to further investigate the unexplored role of integrin a6 in the MES subtype, especially in consideration of the fact that the cellular in vitro models used to analyse the role of integrin a6 in GSCs [22,23] were limited to the PN subtype (CD133^HI^, Oligo2^HI^, and SOX2^HI^ cells) [26,27,47,48]. In contrast, the specific role of integrin a6 in MES-GSCs—i.e., Oligo2^LO^/CD133^LO^/CD44^HI^—still remains unclear.

**Figure 1:**
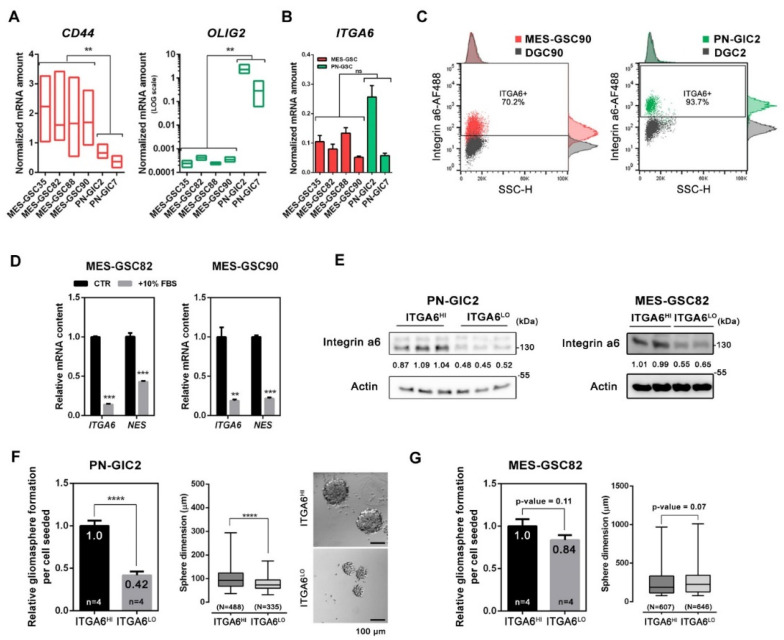
Integrin a6 expression in PN and MES-GSCs. (**A**) Analysis of GSCs from our collection according to the expression of *CD44* and *OLIG2* by q-PCR. The gene expression was calculated using the dCt (unpaired *t*-test with Welch’s correction). (**B**) Expression levels of the *ITGA6* gene across MES-GSCs (depicted in red) and PN-GSCs (depicted in green). Mean expression was calculated with the dCt method (mean ± SEM; *n* = 3; unpaired *t*-test with Welch’s correction). (**C**) Representative flow cytometry analysis of integrin a6 in MES-GSC90 and PN-GIC2 with relative differentiated glioblastoma cells (DGCs) as an internal negative control (Figure S2C). (**D**) *ITGA6* and *NES* mRNA levels after 7 days of differentiation in 10% FBS containing media (mean ± SEM; *n* = 3; unpaired *t*-test). (**E**) Western blot validation of PN and MES-GSCs enriched for integrin a6 high MFI (median fluorescence intensity; HI) or low MFI (LO) via FACS sorting. The values indicated within blots are relative to the densitometric analysis. Numbers indicate the normalized integrin a6 intensity ratio relative to the mean of normalized ITGA6HI samples. (**F**,**G**) Assessment of self-renewal capacity (mean ± SEM; unpaired *t*-test) and sphere size (mean ± SEM; Mann–Whitney test) in PN-GSCs (**F**) and MES-GSCs (**G**) enriched or depleted according to the *ITGA6* expression. Representative micrographs of gliomaspheres formed from PN-GIC2 enriched for *ITGA6* high-expressing and low-expressing cells. The number of independent experiments—*n*, the number of gliomaspheres scored for each condition—N. The values specified within the bar plot indicate the mean relative self-renewal capacity. ** *p* < 0.01; *** *p* < 0.001; **** *p* < 0.0001.

First, we investigated the expression of *ITGA6* in a set of patient-derived GSCs lines. The analysed cultures were maintained as gliomaspheres in serum-free medium and were previously classified as MES-GSCs or PN-GSCs [32] according to the mesenchymal index [26]. The levels of *CD44* and *OLIG2* were used as bona fide markers for MES and PN subtypes, respectively (Figure 1A) [26,27,28,29]. The MES/PN classification obtained was further validated by quantification of *PROM1* (protein name: CD133) transcription level, which is more prevalent in the PN subtype (Figure S2A) [26,27,47]. Consistent with previous observations [27], GSCs bearing Oligo2^HI^/CD44^LO^ PN signature tended to form tightly joined gliomaspheres, whereas Oligo2^LO^/CD44^HI^ MES-GSCs formed irregular grape-shape spheres which tended to be bigger in size (Figure S2B). Within our set of GSCs patient-derived cell lines (four MES-GSCs and two PN-GSCs), *ITGA6* transcriptional levels were comparable and equally high, thus suggesting the relevance of integrin a6 in our MES-GSCs cultures as well (Figure 1B). Flow cytometry highlighted a unique population of integrin a6-expressing cells in PN-GIC2, MES-GSC82, MES-GSC88, and MES-GSC90 (Figure 1C). Paired differentiated GBM cells (DGCs) obtained from same-patient specimens and stained for integrin a6 showed no relevant expression at flow cytometry (Figure 1C). Although PN-GIC2 displayed a higher mean fluorescence intensity and a greater percentage of integrin a6-positive cells (≈94%), the three MES-GSCs still showed a notable portion of cells expressing integrin a6 (mean ≈74%; Figure 1C and Figure S2C).

Given the demonstrated association between integrin a6 and stemness in CD133^HI^ PN settings [22], we wondered whether the differentiation state would also inhibit *ITGA6* expression in the MES subtype. Nestin (*NES*) level was quantified along with *ITGA6* expression as a GBM stemness marker [5], which characterizes GSCs irrespective of their molecular subtype, as demonstrated by previously published data (Figure S2D) [26]. MES-GSC82 and MES-GSC90 were therefore differentiated in serum-containing media and integrin a6 protein level was analysed at 7 days. *ITGA6* expression strongly decreased upon differentiation stimuli together with *NES* expression (Figure 1D) and was totally undetectable in differentiated glioblastoma cells (DGCs) derived from the same post-surgical specimen (Figure S2C) [32]. 

To sum up, our findings demonstrate that *ITGA6* is expressed in both PN and MES GBM subtypes and derived GSCs and is associated with a dedifferentiated status. 

### 3.2. Integrin a6 Supports Stemness in PN-GSCs, but Not in MES-GSCs

As previously shown, *ITGA6* knockdown in Olig2^HI^ PN-GSCs results in abrogation of stem-related features, such as self-renewal reduction and proliferation arrest [22]. To explore whether integrin a6 regulates the same cellular functions in MES-GSCs, we compared FACS-sorted ITGA6^HI^ and ITGA6^LO^ subpopulations (Figure 1E) enriched following the gating strategy specified in Figure S2E. Cells were seeded at single-cell clonal density and the capacity to form gliomasphere, and their diameter were evaluated. Gliomasphere-forming capacity and variation in sphere size roughly reflect stem cell frequency/stem cell potential and proliferation rate, respectively [49,50,51,52,53]. Consistently with published data on PN-GSCs [22], PN-GIC2-ITGA6^LO^ showed a significant reduction in clonogenic capability and sphere size (Figure 1F). In particular, the capacity of PN-GIC2-ITGA6^HI^ to give rise to gliomaspheres was more than double than that of PN-GIC2-ITGA6^LO^, and the mean diameter was 21.65% larger (PN-GIC2-ITGA6^HI^: 100.7 ± 1.9 µm; PN-GIC2-ITGA6^LO^: 78.9 ± 1.5 µm). However, surprisingly, no significant difference was seen in the MES-GSC82 subtype in regard to self-renewal capacity and sphere size (Figure 1G; MES-ITGA6^HI^: 255.7 ± 7.4 µm; MES-ITGA6^LO^: 257.2 ± 6.1 µm). Moreover, another MES-GSCs (MES-GSC90) culture was tested and ITGA6^LO^ did not display reduced sphere size or capacity to form gliomaspheres (Figure S2F). These findings indicated that integrin a6 was required for self-renewal and proliferation in GSCs bearing PN background, but not in MES-GSCs.

**Figure 2:**
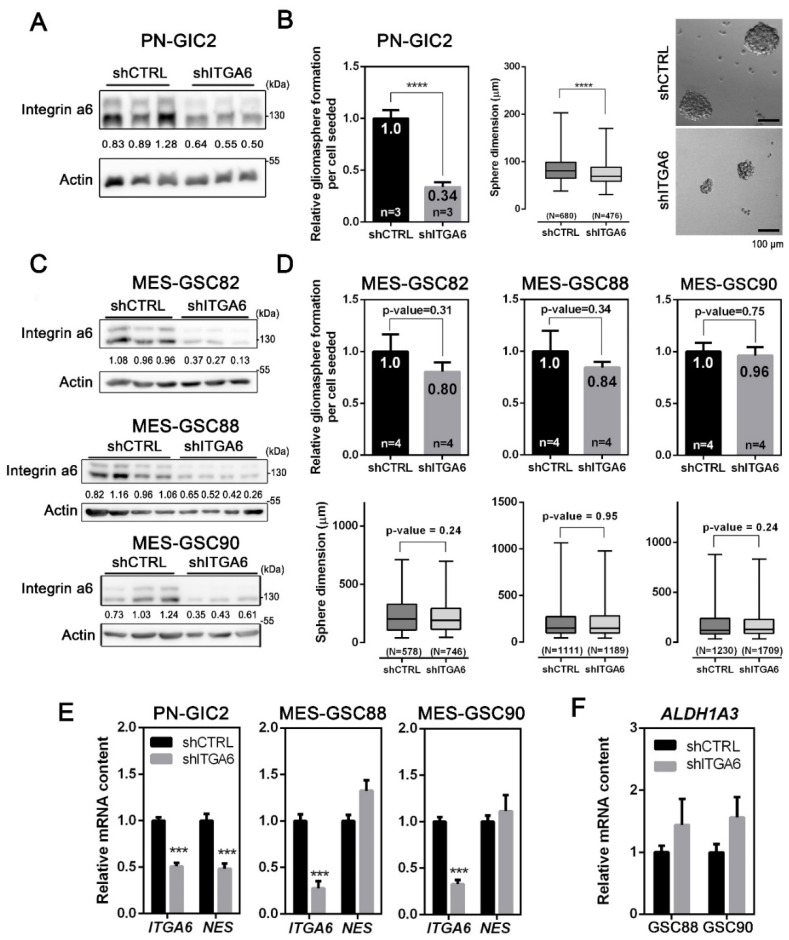
Silencing of integrin a6 in MES-GSCs does not have an impact on stemness and self-renewal. (**A**,**C**) Representative Western blot of the lentiviral-based shRNA silencing of *ITGA6* in PN-GIC2 (**A**) and MES-GSCs (**C**). The values indicated within blots are relative to the densitometric analysis. Numbers indicate the normalized integrin a6 intensity ratio relative to the mean of normalized shCTRL samples. (**B**) Assessment of self-renewal capacity (mean ± SEM; *n* = 3; unpaired *t*-test) and sphere size (mean ± SEM; *n* = 3; Mann–Whitney test) in PN-GSCs silenced for *ITGA6*. Micrographs from representative fields of gliomaspheres (scale bar = 100 µm). (**D**) Self-renewal capacity (mean ± SEM; *n* = 4; unpaired *t*-test) and sphere size (mean ± SEM; *n* = 4; Mann–Whitney test) in MES-GSCs silenced for *ITGA6*. The number of independent experiments—*n*, the number of gliomaspheres scored for each condition—N. The values specified within the bar plot indicate the mean relative self-renewal capacity. (**E**) Transcript levels of *ITGA6* and *NES* detected by means of qPCR in shITGA6 PN-GSCs and MES-GSCs (mean ± SEM; *n* = 3; unpaired *t*-test). (**F**) Relative transcript amount of *ALDH1A3*, a specific MES stem cell marker, in shITGA6 MES-GSCs (mean ± SEM; *n* = 3; unpaired *t*-test). *** *p* < 0.001; **** *p* < 0.0001.

Next, we decided to stably silence the *ITGA6* expression via a lentiviral-based shRNA to better understand its role in GSCs (Figure S3A). After puromycin selection, the obtained gliomasphere cultures displayed a reduced amount of *ITGA6* (Figure 2A,C and Figure S3B). Consistently with the comparison between PN-GIC2-ITGA6^HI^ and PN-GIC2-ITGA6^LO^ (Figure 1G), *ITGA6* silencing in PN-GIC2 reduced the clonogenic potential to 33.84% (Figure 2B) and the spheres formed were 12.85% smaller (PN-GIC2-shCTRL: 86.0 ± 1.1 µm; PN-GIC2-shITGA6: 75.0 ± 1.0 µm). Notably, integrin a6 was essential for the survival of the PN-GIC2, as the *ITGA6* silencing precluded more than two passages in culture. Our results confirmed the previously shown strong impact of integrin a6 on proliferation and stemness in PN settings [22]. Conversely, and consistent with our own findings on MES-ITGA6^HI^ and MES-ITGA6^LO^ (Figure 1G), three different *ITGA6*-silenced MES-GSCs did not display a significant effect on gliomasphere-forming capacity and proliferation (Figure 2D).

To further prove that integrin a6 indeed has an impact on stemness in the PN but not in the MES setting, we evaluated the effect of *ITGA6* downregulation on the expression of a putative stem-cell marker. Silencing of *ITGA6* reduced the expression of *NES* in the PN-GIC2 cells (Figure 2E). In contrast, no effect was associated with *ITGA6* silencing in tested MES-GSCs (Figure 2E). Importantly, no variation was detected for *ALDH1A3,* a recognized specific marker for MES-GSCs [27] (Figure 2F). 

Taken together, our results demonstrate that integrin a6 has no impact on stemness in MES-GSCs, while also corroborating the role of integrin a6 in PN-GSCs stemness [22,23,54]. Accordingly, we decided to further explore the functional role of integrin a6 in MES-GSCs, which, to our knowledge, had not yet been investigated. 

### 3.3. Downregulation of Integrin a6 Affects DNA Damage Repair and Cell Cycle in MES-GSCs

**Figure 3:**
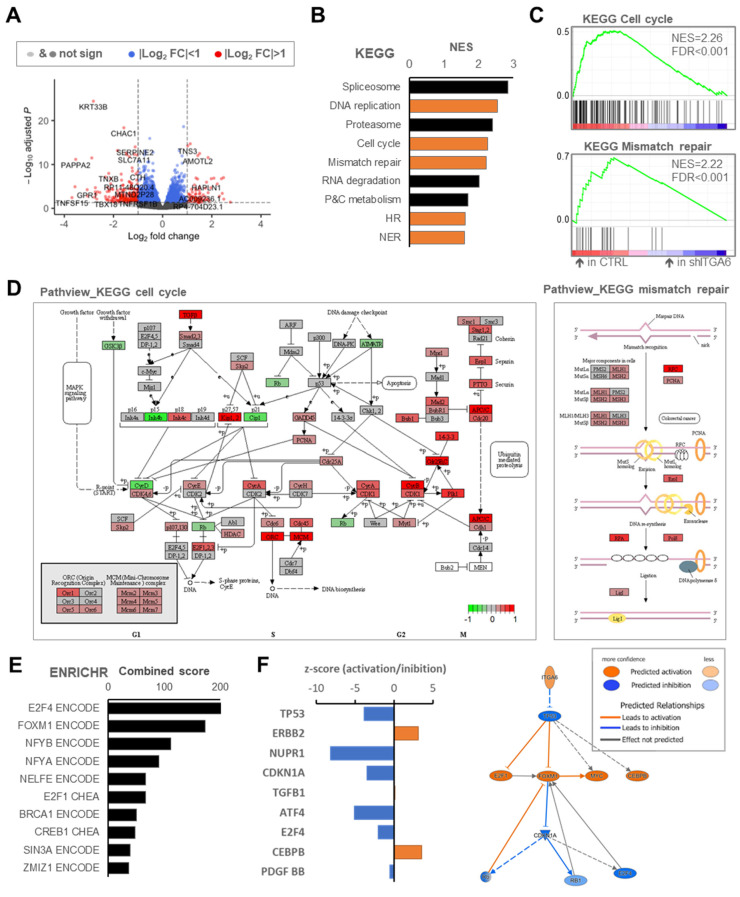
Pathway enrichment analysis following integrin a6 inhibition in MES-GSCs. (**A**) An enhanced volcano plot of differentially expressed genes according to log2(FC) and –log10(*p*-value) within the comparison of CTRL vs. shITGA6. (**B**) Top 9 gene-sets resulting from GSEA as for KEGG tool. The most relevant processes associated with DNA repair dynamics are highlighted in orange colour: DNA replication, mismatch repair, cell cycle, HR, and NER. Used acronyms: P&C metabolism, porphyrin and chlorophyll metabolism; HR, homologous recombination; NER, nucleotide excision repair. (**C**) GSEA plot for the indicated KEGG pathways. (**D**) Pathview KEGG maps of cell cycle and mismatch repair pathway displaying colour variation according to differentially expressed genes (see Figure S4A for KEGG DNA replication). (**E**) Top modulated transcription factor networks as for ENRICHR tool showing the signature related to *E2F4* and *FOXM1*. (**F**) IPA key upstream regulators inhibited (blue colour) or activated (orange colour) in the CTRL cells vs. shITGA6 and their predicted relationships.

To better clarify the role of integrin a6 in the MES context, we performed RNA-Seq analysis of MES-GSCs (MES-GSC90) upon *ITGA6* silencing. Transcriptomic analysis revealed 308 differentially expressed genes with the fold change (FC) < −2 or >2 (Figure 3A; adjusted *p* < 0.05).

Differentially expressed transcripts between shITGA6 and shCTRL samples were then examined to identify biologically relevant clusters. We performed gene set enrichment analysis (GSEA) within the curated KEGG pathways. As expected, the silencing of integrin a6 induced a consistent modulation of extracellular matrix components and interactors (Figure S4A). Moreover and surprisingly, among the top modulated gene sets, we detected pathways related to DNA damage repair and to cell-cycle regulation (Figure 3B,C,D and Figure S4B). Similar results were obtained with GSEA hallmarks gene sets. Indeed, MES-GSC90-shCTRL was found to be significantly enriched in G2/M checkpoint and DNA repair gene sets (Figure S4C,D). 

Next, we investigated enrichment in transcription factor ontologies. We found that E2F-4 and forkhead box protein M1 (coded by *FOXM1*) were the transcription factors most likely to be involved in the transcriptomics changes upon *ITGA6* downregulation (Figure 3E and Figure S4E). Both transcription factors have been implicated in cell cycle regulation [55,56], and FOXM1, in particular, has been associated with enhanced sensitivity to genotoxic stress in GBM [57,58,59,60]. Indeed, most of the FOXM1 and E2F4 direct transcriptional targets found to be significantly upregulated in shCTRL cells (Figure S4F,G) are involved in cell cycle regulation and G2/M checkpoint pathways after DNA damage [61,62,63,64,65]. Analysis of TCGA datasets in glioma patients confirmed significant positive correlation of *ITGA6* expression with *CDK1*, *CDK4*, *PCNA,* and *FOXM1* transcript levels (Figure S5A,B). Based on these data, high levels of integrin a6 potentially convey resistance to DNA damaging therapies in MES-GSCs. 

To gain further insights into the biological value of the *ITGA6* expression in the mesenchymal setting, we performed Ingenuity Pathway Analysis (IPA). Interestingly, IPA predicted that *ITGA6* induces the *TP53* pathway inhibition, thus activates *FOXM1* and inhibits *CDKN1A* (p21) and *E2F4* (Figure 3F). As a consequence, the molecular alterations happening in *ITGA6*-silenced cells convey increased cell morbidity, senescence, and necrosis (Figure S4H). 

Recently, the inhibition of integrin a6 has been described as negatively impacting on the FOXM1 regulatory node in an ERK-dependent manner in Oligo2^HI^ GSCs (PN-GSCs) [54]. However, in our Oligo2^LOW^ CD44^HI^ GSCs (MES setting), no inhibition was detected in this signalling axis upon silencing of *ITGA6* (Figure S5C). Moreover, no perturbation of ERK phosphorylation was detected following *ITGA6* silencing (Figure S5D). Finally, consistent with our results in Figure 2F, stem-related genes were not affected by *ITGA6* inhibition (Figure S5E). 

Taken together, our results suggest that integrin a6 regulates pathways crucial for cell cycle and DNA damage repair in GSC-MES, and its inhibition may sensitize tumoral cells to DNA damaging therapeutic stress.

### 3.4. Integrin a6 Is Critical for MES-GSCs Sensitivity to Ionizing Radiation

**Figure 4:**
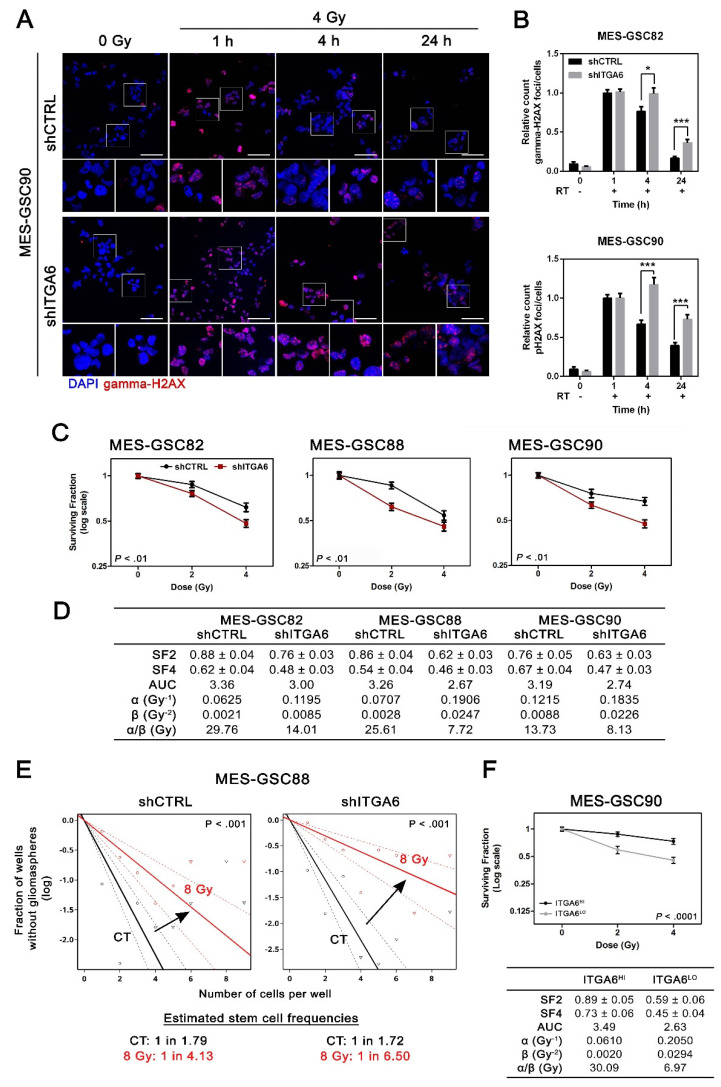
Inhibition of integrin a6 expression in MES-GSCs triggers radiosensitivity. (**A**,**B**) Gamma-H2AX foci decay after IR-induced DNA damage. (**A**) Representative microsections of the gamma-H2AX foci detected in MES-GSC90 by immunofluorescence (scale bar = 50 µm). Gamma-H2AX foci were stained in red, while nuclei were counterstained with DRAQ5. Smaller frames display the same sections at higher magnification. (**B**) Absolute quantitation of gamma-H2AX foci after a single fraction of 4 Gy (mean ± SEM; *n* = 3; unpaired t-test). A minimum of 10 fields per condition reaching a minimum of 70 cells in total were analysed (*n* = 3). (**C**) Survival curves of MES-GSCs obtained for control (shCTRL) and *ITGA6*-inhibited cultures (shITGA6) following RT (*n* = 4). Two-way ANOVA reported within each plot. (**D**) Linear quadratic model and survival curve parameters to quantify radiation sensitivity. SF2 and SF4 are indicated as mean ± SEM. SF2, surviving fraction at 2 Gy; SF4, surviving fraction at 4 Gy; AUC, area under the curve. (**E**) In vitro extreme limiting dilution assay to test radiation sensitivity. It revealed the sphere formation frequencies of control and shITGA6 MES-GSC88 untreated or 8 Gy irradiated. Pairwise test *p*-value reported within each single plot. (**F**) (**top**) Survival curve of MES-GSC90 obtained for ITGA6HI and ITGA6LO cultures following RT (*n* = 3; two-way ANOVA). (**bottom**) Linear quadratic model and survival curves parameters to quantify radiation sensitivity. * *p* < 0.05; *** *p* < 0.001.

To corroborate that silencing of *ITGA6* impacts on DNA damage response in MES-GSCs, we quantified the magnitude of DNA double-strand breaks (DSB) accumulation, via the phosphorylated status of the histone H2AX (gamma-H2AX), and its resolution over time [66]. The effect of integrin a6 downregulation on DNA damage repair was not significant in basal conditions, despite a slight tendency of shITGA6 GSCs toward accumulating a larger number of gamma-H2AX positive foci. However, when shITGA6 MES-GSC82 and shITGA6 MES-GSC90 were exposed to ionizing radiation following a treatment regimen mimicking a clinically relevant radiotherapy setting (RT), a significant reduction in the capacity to repair DNA damage was detected. Specifically, MES-GSCs were irradiated, and foci formation and decay were analysed 1, 4, and 24 h afterwards. Interestingly, *ITGA6* downregulation significantly reduced the capacity of MES-GCS82 and MES-GSC90 to repair DNA damage, with a delayed decay in gamma-H2AX foci resolution 4 and 24 h after RT exposure (Figure 4A,B). Thus, *ITGA6* inhibition downregulates the DNA damage repair machinery in MES-GSCs and significantly alters their capacity to recover from induced DSB.

In order to assess whether the inhibition of DNA damage repair consequent to integrin a6 downregulation may play a role in a clinically relevant context, we challenged *ITGA6*-silenced MES-GSC82, MES-GSC88, and MES-GSC90 with a fractionated protocol of RT. The radiosensitivity was evaluated by means of a clonogenic assay at 2 and 4 Gy (Figure 4C). Downregulation of *ITGA6* clearly enhanced radiosensitivity in all the MES-GSCs tested. For every survival curve, the surviving fractions (SFs) were calculated at each dose along with the area under the curve (AUC) (Figure 4D). In multiple shITGA6 MES-GSCs tested, SFs and the AUC obtained were smaller, indicating a more radiosensitive background. To gain more insights into the radiobiological value of the test performed, the curves were interpreted using the linear quadratic model, a commonly used mathematical model in clinical radiation oncology [35,36,38,67,68,69]. shITGA6 cells displayed higher α- and β-values, indicating increased radiosensitivity and impaired capacity to repair sublethal DNA damage (Figure 4D). In addition, lower α/β ratio of shITGA6 suggested an enhanced sensitivity to fractionated doses [38,69,70].

Finally, to address whether genotoxic stress in combination with *ITGA6* silencing inhibits stemness, we estimated the fraction of cells bearing stem cell potential and self-renewal capacity via extreme limiting dilution assay (ELDA) [39]. As expected, and in line with Figure 2, *ITGA6* silencing did not change the GSCs proportion in non-irradiated cultures (shCTRL CT vs. shITGA6 CT), while it displayed a significant impact over the capacity to retain stemness compared with irradiated cells (Figure 4E). The reduction of stem cell frequency was significantly greater in all three irradiated *ITGA6*-silenced MES-GSCs cultures (Figure S6A). Though the intrinsic stemness of MES-GSCs was not altered by integrin a6 downregulation, higher *ITGA6* levels after irradiation correlated with better capacity to maintain stem cell pool. Similar results were obtained analysing MES-GSC82 and MES-GSC90 FACS-sorted ITGA6^HI^ and ITGA6^LO^ (Figure 4F and Figure S6B). Indeed, cells with greater expression of integrin a6 were characterized by a higher proportion of stem cells only when irradiated (Figure S6C). This ruled out the possibility that the observed effects were induced by side effects of the silencing.

Furthermore, the molecular insights we obtained on the role of integrin a6 in MES-GSCs allowed us to focus on its relevance for glioma patients’ outcome. In particular, the 12 genes most upregulated by the expression of *ITGA6* in MES-GSCs correlated negatively with glioma patients’ survival, as shown by the Cox proportional hazards regression model (hazard ratio, 4.4; 95% confidence interval, 3.31–5.86; *p* = 1.89 × 10^−24^; Figure S7).

Therefore, MES-GSCs with greater expression of integrin a6 display a more efficient DNA damage response machinery and are hence more radioresistant.

## 4. Discussion

Integrins are a family of adhesion molecules driving cell-to-cell and cell–ECM communication. These transmembrane proteins are involved in various cellular processes, including cell survival, proliferation, migration, invasion, and angiogenesis, and consequently, their functions are expected to potentially support tumour development [71,72,73,74]. Targeting integrins is an attractive goal as these adhesion molecules are involved in crucial aspects of malignant progression. Furthermore, integrin expression patterns in neoplastic lesions differ from those of non-neoplastic tissues, thus allowing selective targeting of tumoral cells. 

In recent years, a promising peptide, named Cilengitide, was developed to target and selectively inhibit integrin heterodimers av/b3 and av/b5. Despite encouraging preclinical studies in GBM mouse models and early phase trials [75,76], the use of Cilengitide did not show significant clinical improvement when combined with standard therapies [77,78]. 

Integrin a6 has been described in GBM as a GSCs marker capable of selectively enriching for GSCs independently of CD133 expression and sustaining GCS self-renewal, proliferation, and tumour initiating capacities [22]. Since then, the biological role of integrin a6 has been extensively investigated in GSCs characterized by expression of Oligo2 and CD133 markers, among others [22,23,54]. These markers are broadly used as part of the signature defining the PN subtype [27,28,47]. Given the lack of knowledge on the role of integrin a6 in GSCs belonging to the MES subtype and the high expression of this marker in GSC-MES [32] and in bulk TCGA-MES GBM (Figure S1), we explored its relevance in the PN versus MES subtype. Importantly, the study of key pathways may offer a comprehensive evaluation of the molecular subtypes coexisting in the lesion, especially in light of its heterogeneous biology.

We observed that, differently from PN cells where it supports cell proliferation and stemness, integrin a6 did not drive cancer stem cell maintenance and proliferation in MES-GSCs (i.e., Oligo^LOW^ CD44^HI^ patient-derived gliomaspheres).

Computational analysis indicated that *ITGA6* downregulation in MES-GSCs modulates pathways crucial for DNA replication, mismatch repair, and cell cycle regulation, engaging particularly with transcription factor FOXM1. Most of the target genes of FOXM1 found altered by *ITGA6* silencing, when inhibited in unperturbed cells, lead to a simple delay in the mitotic entry [62]. However, when DNA damage is therapeutically induced, the inefficient machinery may lead to an incorrect or absent release from the mitotic arrest [79].

Despite the limitations of the transcriptomic analysis conducted on a single cell line (MES-GSC90), further functional validations were consistent across the different MES-GSCs analysed.

Of note, *ITGA6* expression inhibition effectively impaired DNA damage repair machinery in MES-GSCs leading to a significant delay in foci recovery dynamics.

MES-GSCs expressing lower amounts of integrin a6 were consistently characterized by increased radiosensitivity and by a reduced capacity to retain stemness following RT. 

Integrin a6 involvement in radioresistance mechanisms has been previously ascribed to its control over the AKT/ERK pathway [80] and ZEB1/ERK transcription factor [23,54], in breast cancer and GBM, respectively. Nevertheless, we found that the downregulation of *ITGA6* expression in GSCs bearing MES signature had little impact on these targets. 

Cellular heterogeneity is a hallmark of GBM and is closely related to the presence of GSCs at different transcriptional states [45,81,82]. In addition, single-cell RNA-seq revealed the presence of distinct GSCs profiles in the same tumour [30]. Proneural to mesenchymal transition (PMT) can be induced upon selective pressure of different factors and treatments during GBM progression [26,28,83]. The results from our study suggest that targeting integrin a6 may reduce the number of PN-GSCs and overcome radioresistance in MES-GSCs within the same tumour. This may be of particular interest in order to counteract the radiation-induced PMT and the selection of radioresistant subpopulations. 

Notably, the molecular insights we acquired on the role of integrin a6 across different subtypes highlight its relevance for GBM outcome. However, further investigations on a larger cohort of GSCs to enforce and validate present findings are recommended.

Taking into consideration that the current standard of care in GBM [84] uses mainly DNA-damaging therapies—radiotherapy and the DNA methylating agent temozolomide—the impact of integrin a6 on DNA damage repair may represent a key turning point. Irrespective of the molecular profile of GSCs, perturbation of *ITGA6* leads to weaker and more fragile cells which limits the capacity to cope with genotoxic stress. Importantly, *ITGA6* silencing was able to enhance sensitivity to radiation in a MES-GSCs culture line that demonstrated a striking radioresistant phenotype in previous studies [32]. Thus, integrin a6 inhibition may represent a promising strategy to overcome GBM resistance to radiotherapy, even in the most refractory cases. However, many are the pitfalls to finding an effective and safe therapy capable of inhibiting integrin a6 in vivo, since the functional blocking antibody now available may potentially cause important side effects if systemically administrated, due to the fact that integrin a6 is broadly expressed in epithelium and intestine. One possible resource is local inoculation of inhibitory drugs directly in the surgical cavity before irradiation.

Despite great efforts within the scientific community [85], the diagnostic strategies for GBM still lack effective biomarkers at the moment, and pathologists rely on a “layered” and “integrated diagnosis” [86]. Since 2016, glioblastomas are internationally classified based on the IDH1/2 status and histopathological features (nuclear atypia, cellular pleomorphism, microvascular proliferation, and/or necrosis) [87]. Given the results obtained in the present and previous studies [22], we can hypothesize integrin a6 expression as a potential prognostic marker for GBM patients, irrespectively of the molecular subtype. However, further investigations on a larger cohort of GSCs to enforce and validate present findings are recommended. Indeed, at this stage, functional implications of the integrin a6 still remain speculative and warrant further research.

An important final remark on the microenvironment where integrin a6 may be relevant within the whole GBM: We know that integrin a6 is a receptor for laminins [74]. Within the brain, laminins are mainly localized in the vascular basement membrane around blood vessels [88,89,90]. This makes our discoveries very relevant for a better understanding of the tumour topology upon treatment. We can indeed envision a condition where GBM cells that express more integrin a6 are more MES-like, radioresistant, and localized around blood vessels—as previously shown for MES-like cells [91,92]—thus making the perivascular niche an important hub for resistance to therapy [6,93,94,95,96]. Therefore, another potential strategy to reduce the effects of integrin a6 may be reducing the movement of GBM cells towards pre-existing blood vessels, also called vessel co-option [97,98]. This therapeutic strategy may be potentially safer than directly targeting integrin a6.

Taken together, the findings presented here highlight the crucial relevance of integrin a6 in the radioresistance of MES-GSCs and suggest that integrin a6 may represent an attractive target to enhance GBM radiocurability.

## 5. Conclusions

In the current study, we observed a different role of integrin a6 in GSCs across proneural and mesenchymal signature. The data obtained show that *ITGA6* in PN-GSCs tightly regulates proliferation and stemness-related features. Despite the limited number of GSCs investigated in the present study, the transcriptomic analysis suggests that silencing of *ITGA6* in MES-GSCs weakens the cell-cycle pathway and DNA damage response machinery. Importantly, all silenced MES-GSCs cultures tested showed an increased sensitivity to genotoxic stress, such as ionizing radiation. These data highlight the importance of discriminating between transcriptional subtypes when studying the heterogeneous biology of GBM. Lastly, we identified an attractive mechanism that may harm both GSCs subtypes and potentially controls tumour relapse following conventional treatment.

## Data Availability

The RNA-sequencing data presented in this study are available at GEO reference GSE178260.

## Supplementary Materials

The following are available online at https://www.mdpi.com/article/10.3390/cancers13123055/s1: Figure S1: Integrin a6 expression across publicly available repositories, Figure S2: PN and MES-GSCs characterization according to *ITGA6* expression, Figure S3: Validation of integrin a6 silencing in GSCs, Figure S4: Computational analysis following silencing of a6 in a mesenchymal setting, Figure S5: Integrin a6 expression correlates with top identified markers in GBM patients and in a mesenchymal setting does not impact on pathways identified for proneural GSCs, Figure S6: Assessment MES-GSCs stemness and radioresistance following a6 expression inhibition or enrichment, Figure S7: Kaplan-Meier survival curves in glioma TCGA publicly available database. Full website blot Figures can view at the supplementary file.
