# Supplementary material for: Dual Role of Integrin Alpha-6 in Glioblastoma: Supporting Stemness in Proneural Stem-Like Cells While Inducing Radioresistance in Mesenchymal Stem-Like Cells
Source: Cancers (Basel). 2021 Jun 19;13(12):3055. doi: 10.3390/cancers13123055 (PMC8235627; doi:10.3390/cancers13123055)
Supplement: Supplementary file 1 [file cancers-13-03055-s001.zip › cancers-1209350-supplementary/revised supp+WB/cancers-1209350 revised supp.pdf]

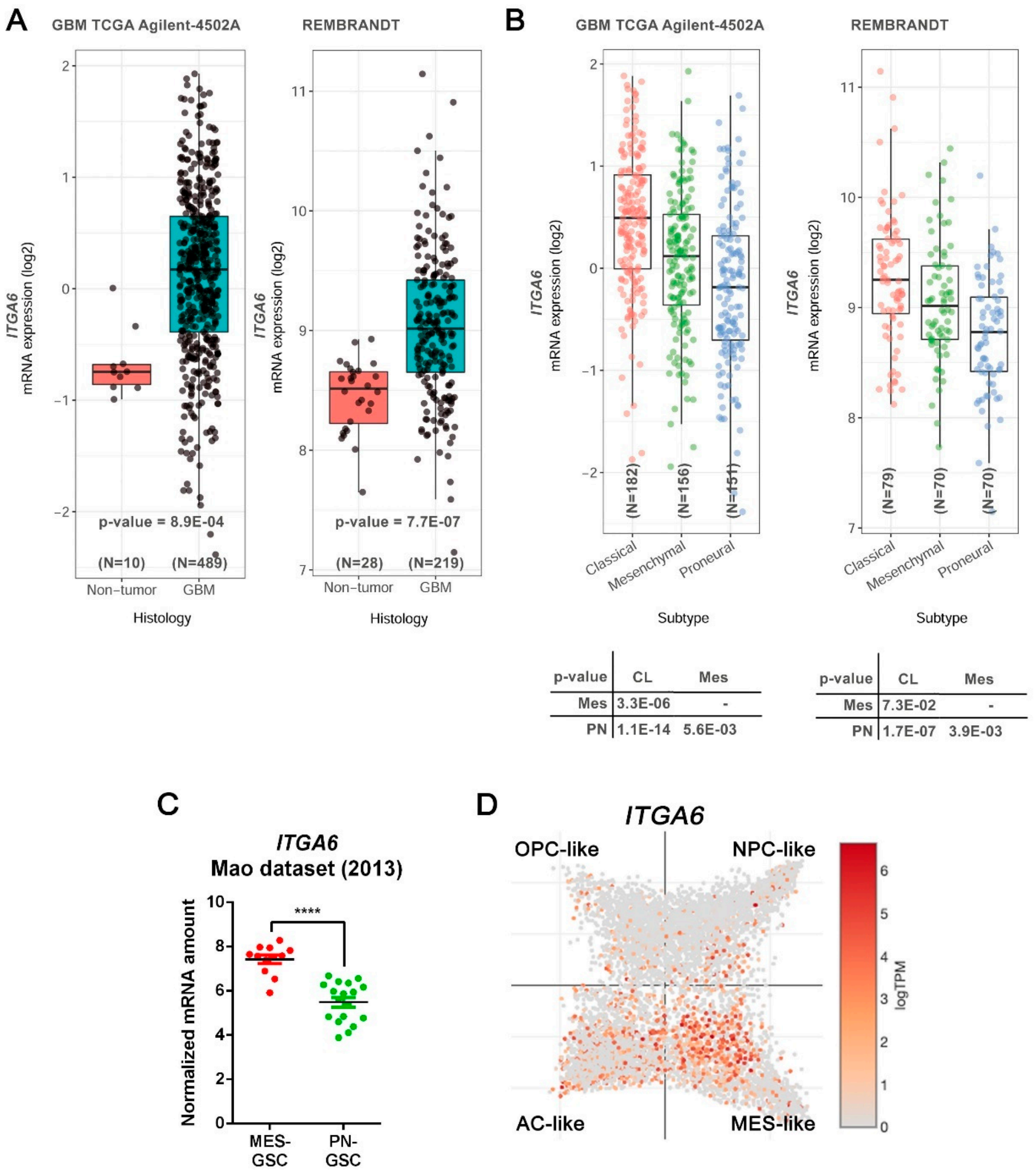

Figure S1. Integrin a6 expression across publicly available repositories. (**A**) *ITGA6* expression in GBM bulk specimens vs non-tumoral samples according to TCGA collection (left panel) and Rembrant collection (right panel). Displayed p-values are calculated following pairwise comparisons between group levels with corrections for multiple testing (p-values with Bonferroni correction) as calculated by GlioVis portal. The number of included samples is specified in brackets following N – uppercase. (**B**) *ITGA6* expression in GBM specimens stratified on the basis of the mRNA subtype profile belonging to the TCGA collection (left panel) and the Rembrant collection (right panel). Displayed p-values are calculated following pairwise comparisons between group levels with corrections for multiple testing (p-values with Bonferroni correction) as calculated by GlioVis portal. The number of included samples is specified in brackets following N – uppercase. (**C**) In-vitro *ITGA6* expression level of GSC classified as MES-GSC and PN-GSC according to their transcriptomic profile. Publicly available data from Mao [1] (unpaired t-test with Welch's correction; transcript analysed: 11736029_a_at) is reported. (**D**) GBM cell state hierarchy plot as defined by Neftel [3] displaying *ITGA6* expression across diverse states. Oligodendrocyte progenitor-like (OPC-like), neural progenitor like (NPC-like), astrocyte-like (AC-like), and mesenchymal-like (MES-like) states.

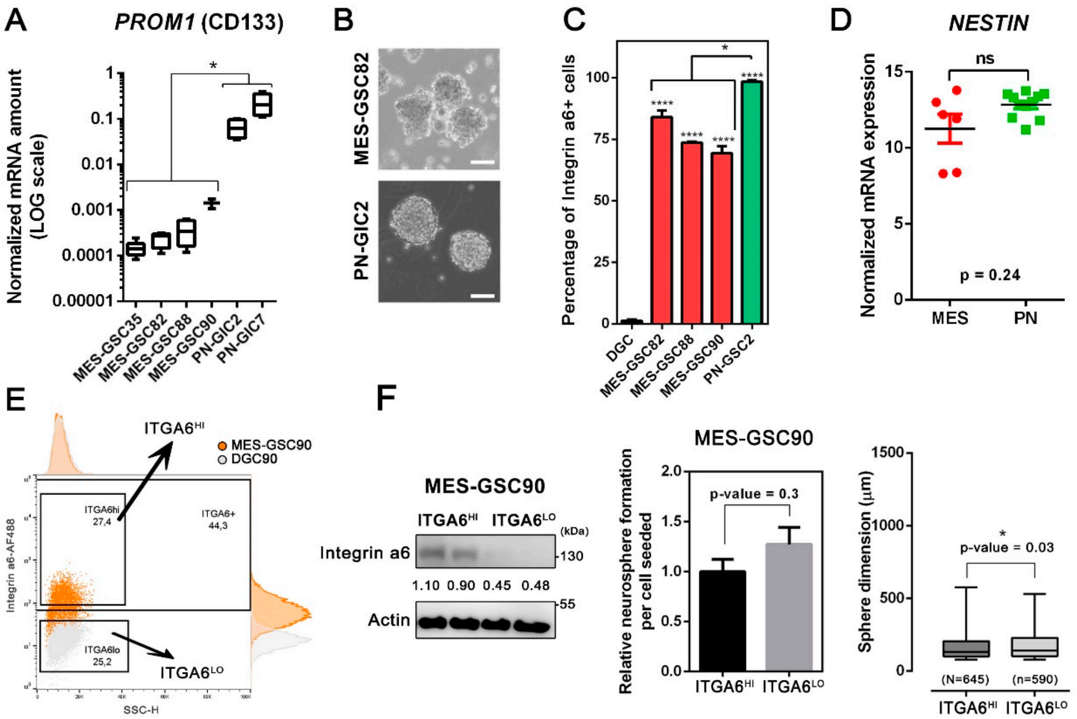

Figure S2. PN and Mes-GSC characterization according to *ITGA6* expression. (**A**) Analysis of PROM1 (CD133) expression in our GSC-collection by q-PCR. The gene expression is calculated using the dCt (Unpaired t-test with Welch's correction). (**B**) Representative micrograph of a GSC culture displaying a mesenchymal signature and a proneural signature. (**C**) Percentage of *ITGA6*-positive cells within GSC collection as detected via Flow-cytometry. Samples of differentiated GBM cells (DGC) are included as internal control of cells not expressing *ITGA6* (mean±SEM; statistic comparison unless otherwise specified is referred to DGC internal control; ordinary one-way ANOVA with Dunnet's correction for multiple comparisons). (**D**) Publicly available data from Bhat dataset [2] (Mann-Whitney test) is reported. (**E**) Gating strategy used to sort ITGA6-HI and ITGA6-LO GSC. Cells from the upper quartile and lower quartile were isolated and sorted. (**F – left panel**) Western blot validation of MES-GSC90 enriched for ITGA6-HI or ITGA6-LO via FACS sorting. The values indicated within blots are relative to the densitometric analysis. Numbers indicate the normalized integrin a6 intensity ratio relative to the mean of normalized ITGA6^{HI} samples. (**F – right panel**) Assessment of self-renewal capacity (mean±SEM; Unpaired t-test) and sphere size (mean±SEM; Mann Whitney test) in Mes-GSC90 enriched or depleted according to *ITGA6* expression. The number of gliomaspheres scored for each condition are indicated after N – upper case.

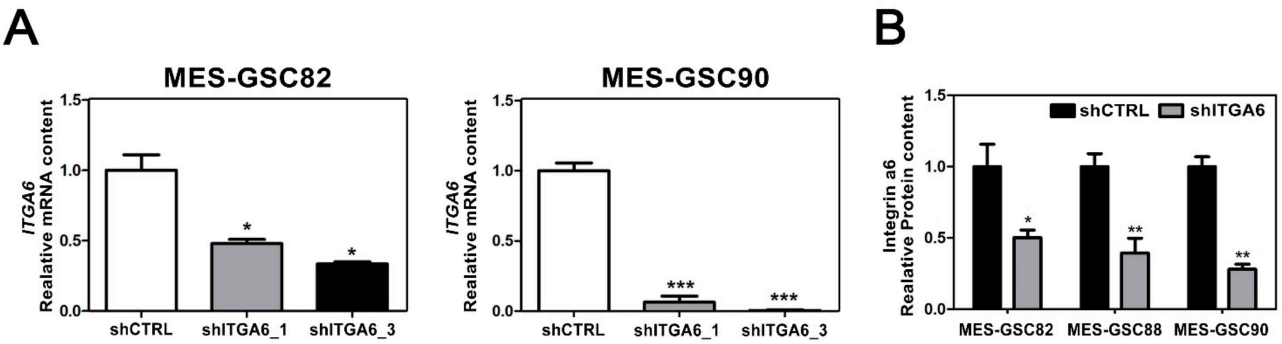

Figure S3. Validation of integrin a6 silecing in GSC. (**A**) Analysis of *ITGA6* expression in MES-GSC82 and MES-GSC90 following *ITGA6* silencing by q-PCR. Two different short-hairpin RNA sequences were tested. The gene expression is calculated using the ddCt (Unpaired t-test). According to the relative silencing capacity obtained, the short-hairpin shITGA6_3 was used for the rest of the experiments. (**B**) Quantification of integrin a6 protein via densitometry of western blot after *ITGA6* silencing with shITGA6_3 (Unpaired t-test).

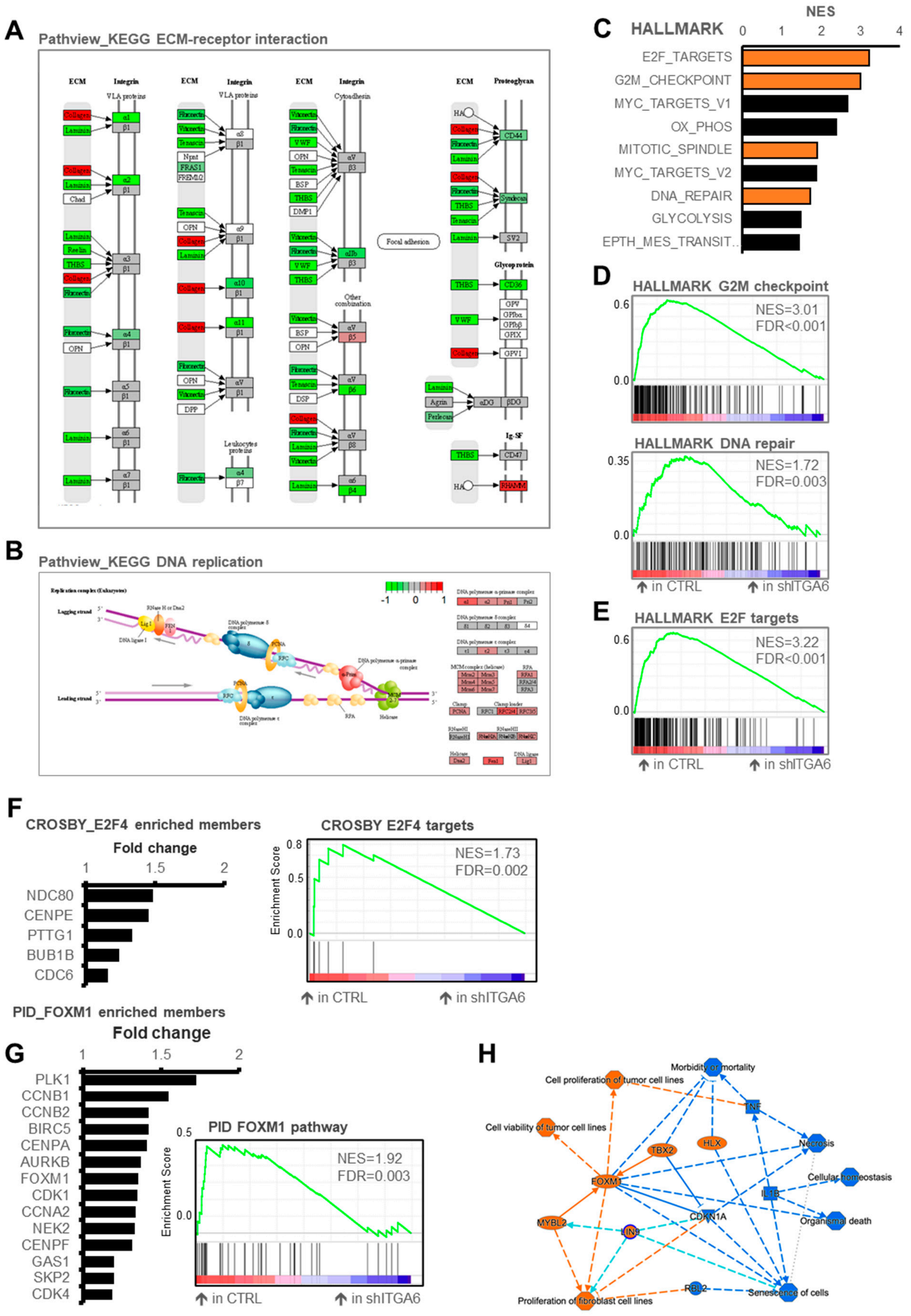

Figure S4. Computational analysis following silencing of integrin a6 in a mesenchymal setting. (**A-B**) Pathview KEGG map of ECM-receptor interaction (**A**) and DNA replication (**B**) displaying colour variation according to differentially expressed genes. (**C**) GSEA Hallmark enrichment showing in orange the most relevant upregulated processes in shCTRL samples and in light-blue the most relevant downregulated gene sets in compare to shITGA6 cells. (**D**) GSEA plot for Hallmark G2M checkpoint and DNA repair.

(**E**) GSEA plot for Hallmark E2F targets. (**F-G**) Top genes associated to E2F4 (**F**) and FOXM1 (**G**) signalling significantly modulated by ITGA6-silencing and their relative GSEA plots. (H) IPA predicted interactions among altered regulators (inhibited: blue-color) or activated: orange-color) in CTRL cells vs shITGA6.

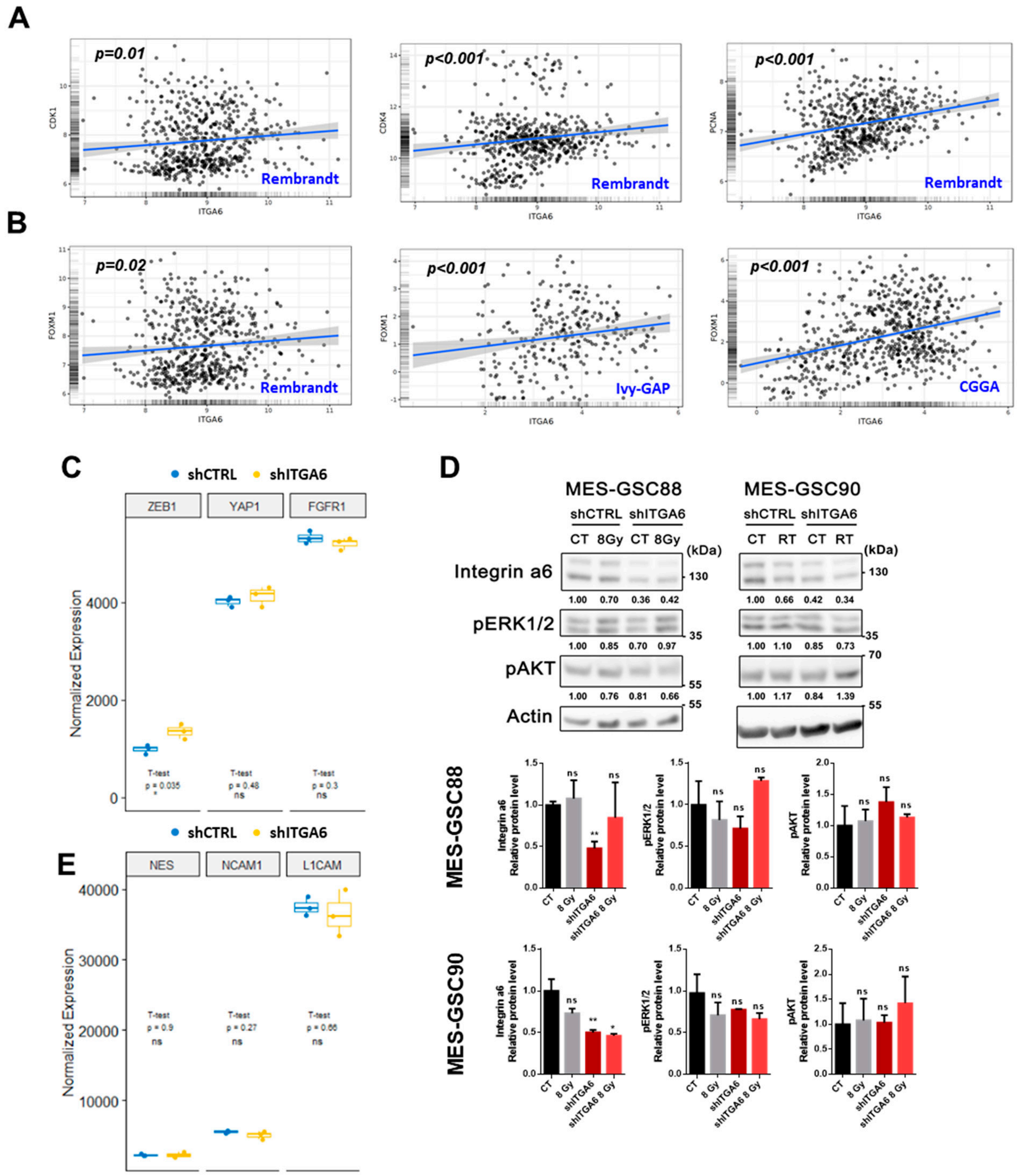

Figure S5. Integrin a6 expression correlates with top identified markers in GBM patients and in a mesenchymal setting does not impact on pathways identified for proneural GSC. (**A**) Correlation plots between *ITGA6* expression and CDK1, CDK4 and PCNA, respectively, within Rembrandt collection. GlioVis calculated two-sided Pearson's product-moment correlation is displayed within each plot. (**B**) Correlation plots between *ITGA6* expression and FOXM1 according to Rembrandt, Ivy-GAP and CGGA collections. GlioVis calculated two-sided Pearson's product-moment correlation is displayed within each plot. (**C**) Expression of putative PN-GSC integrin a6 downstream target as obtained from RNA-seq analysis of MES-GSC90 following *ITGA6* knock-down. The genes analyzed do not display significant variation accordingly to *ITGA6* expression. Conversely, ZEB1 transcript results significantly upregulated in shITGA6 samples. (**D-upper panel**) Representative western blot depicting the absence in ERK1/2 and AKT phosphorylation status perturbation following *ITGA6* silencing in two different MES-GSC. CT refers to unirradiated control samples, whereas 8 Gy samples were collected after 1 hour from the fourth 2 Gy-fraction (total dose: 8 Gy delivered following fractionated schedule; single fraction: 2 Gy every 24 hours). The values indicated within blots are relative to the densitometric analysis of the blot shown. Numbers indicate the relative Beta-Actin normalized integrin a6 intensity ratio in compare to the shCTRL samples. (**D-**

lower panel) Densitometric analysis of three independent experiments was reported. (**E**) Expression of consolidated GSC markers following *ITGA6* knock-down in MES-GSC90 as obtained from RNA-seq analysis.

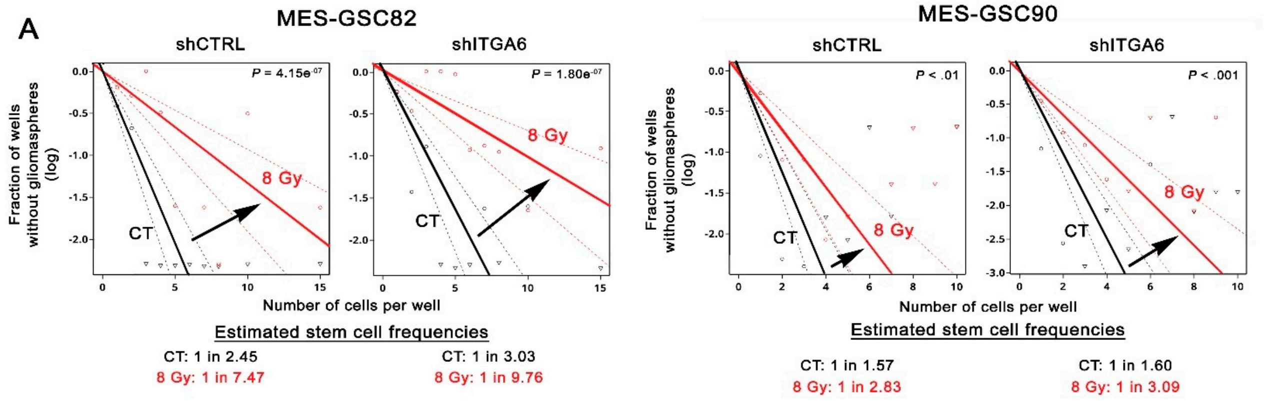

B

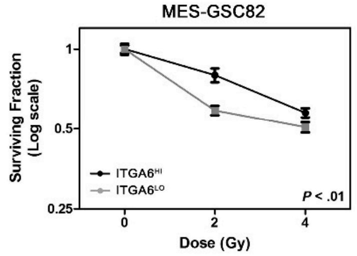

MES-GSC82

	ITGA6^{HI}	ITGA6^{LO}
SF2	0.80 ± 0.05	0.59 ± 0.02
SF4	0.58 ± 0.02	0.51 ± 0.02
AUC	3.17	2.68
α (Gy^{-1})	0.1010	0.2060
β (Gy^{-2})	0.0059	0.0298
α/β (Gy)	17.08	6.92

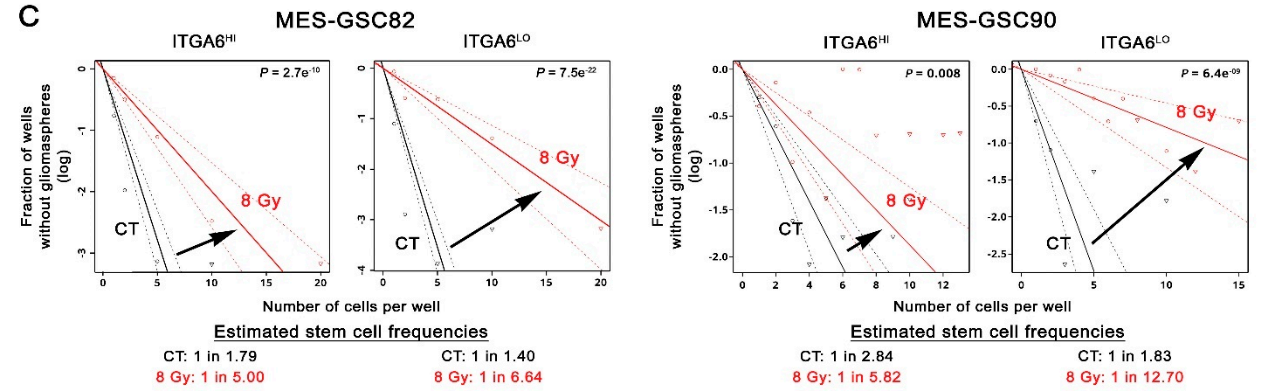

Figure S6. Assessment MES-GSC stemness and radioresistance following integrin a6 expression inhibition or enrichment. (**A**) In vitro extreme limiting dilution assay to test radiation sensitivity of control and shITGA6 MES-GSC untreated or 8 Gy irradiated. Pairwise test p-value reported within each single plot. (**B - left panel**) Survival curves of MES-GSC82 obtained for ITGA6^{HI} and ITGA6^{LO} following RT (n=4). The calculated p-value following two-way ANOVA is reported. (**B - right panel**) Linear quadratic model and survival curves parameters to quantify radiation sensitivity. SF2 and SF4 are indicated as mean ± SEM. SF2, surviving fraction at 2 Gy; SF4, surviving fraction at 4 Gy; AUC, area under the curve. (**C**) In vitro extreme limiting dilution assay of ITGA6^{HI} and ITGA6^{LO} MES-GSC untreated or 8 Gy irradiated. Pairwise test p-value reported within each single plot.

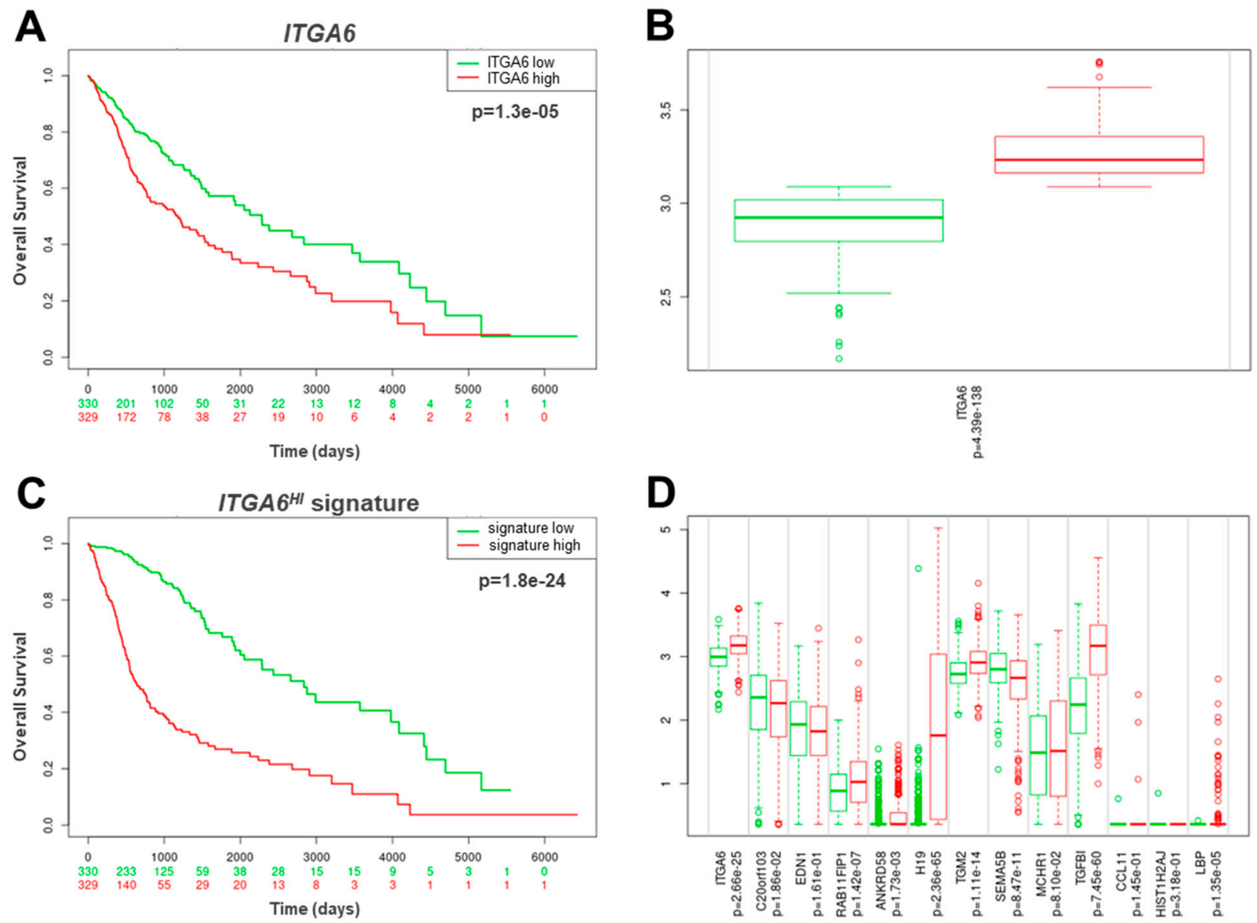

Figure S7. Kaplan-Meier survival curves in glioma TCGA publicly-available database. (**A**) Overall survival by *ITGA6* expression [Hazard ratio (HR) = 1.77; 95% Confidential Interval (95%Confidence Interval=1.37-2.29), p=1.03e-05, Log-Rank test]. (**B**) *ITGA6* expression by risk groups. (**C**) ITGA6^{HI} signature as biomarker [HR=4.41 (95% Confidence Interval=3.31-5.86), p=1.8e-24; Log-Rank test]. (**D**) Relative expression by risk groups of the genes composing the ITGA6^{HI} signature *(ITGA6, C20orf103, EDN1, RAB11FIP1, ANKRD58, H19, TGM2, SEMA5B, MCHR1, TGFBI, CCL11, HIST1H2AJ, LBP).*

Reference

1. Mao, P.; Joshi, K.; Li, J.; Kim, S.-H.; Li, P.; Santana-Santos, L.; Luthra, S.; Chandran, U.R.; Benos, P.; Smith, L.; et al. Mesenchymal glioma stem cells are maintained by activated glycolytic metabolism involving aldehyde dehydrogenase 1A3. *Proc. Natl. Acad. Sci.* **2013**, *110*, 8644–8649, doi:10.1073/pnas.1221478110.
2. Bhat, K.P.L.; Balasubramaniyan, V.; Vaillant, B.; Ezhilarasan, R.; Hummelink, K.; Hollingsworth, F.; Wani, K.; Heathcock, L.; James, J.D.; Goodman, L.D.; et al. Mesenchymal Differentiation Mediated by NF-κB Promotes Radiation Resistance in Glioblastoma. *Cancer Cell.* **2013**; *24*, 331–346.
3. Neftel, C.; Laffy, J.; Filbin, M.G.; Hara, T.; Shore, M.E.; Rahme, G.J.; Richman, A.R.; Silverbush, D.; Shaw, M.L.; Hebert, C.M.; et al. An Integrative Model of Cellular States, Plasticity, and Genetics for Glioblastoma. *Cell* **2019**, *178*, 835–849.e21, doi:10.1016/j.cell.2019.06.024.
