# Supplementary material for: Dual Role of Integrin Alpha-6 in Glioblastoma: Supporting Stemness in Proneural Stem-Like Cells While Inducing Radioresistance in Mesenchymal Stem-Like Cells
Source: Cancers (Basel). 2021 Jun 19;13(12):3055. doi: 10.3390/cancers13123055 (PMC8235627; doi:10.3390/cancers13123055)

Please uploaded a single PDF file or a zip folder including the original, uncropped and unadjusted images supporting all blot and gel results reported in main article or supplementary materials. Authors should annotate each original image, corresponding to the figure in the main article or supplementary materials, and label each lane or loading order. All experimental samples and controls used for one comparative analysis should be run on the same blot/gel image. The different images should not be spliced together to illustrate the results.

All membranes were blotted together with the Thermo Scientific™ PageRuler™ Plus Prestained Protein Ladder, 10 to 250 kDa (ref. 26620) as reference.

Figure 1 panel E - PN-GIC2 sorted GSC

Lane 1: PN-GIC2 ITGA6^{HI} A (*)
Lane 2: PN-GIC2 ITGA6^{HI} B (*)
Lane 3: PN-GIC2 ITGA6^{HI} C (*)
Lane 4: PN-GIC2 ITGA6^{LO} A (*)
Lane 5: PN-GIC2 ITGA6^{LO} B (*)
Lane 6: PN-GIC2 ITGA6^{LO} C (*)
(*) Lanes displayed in the figure panel

Integrin a6 antibody chemiluminescent signal and relative membrane (exposure time 16 min)

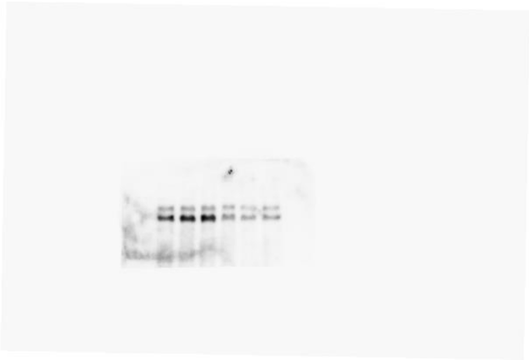

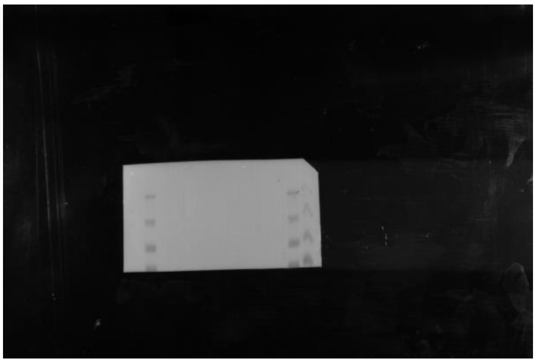

Actin antibody chemiluminescent signal and relative membrane (exposure time 1.5 min) relative to membrane section #2

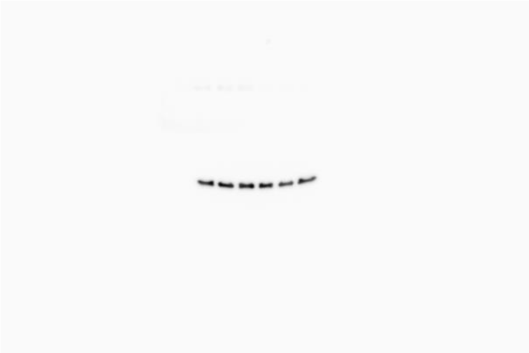

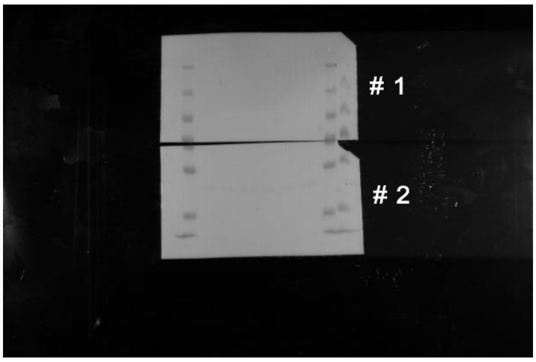

Figure 1 panel E – MES-GSC82 sorted GCS

Lane 1: MES-GSC82 $ITGA6^{HI}$ A (*)
Lane 2: MES-GSC82 $ITGA6^{HI}$ B (*)
Lane 3: MES-GSC82 $ITGA6^{LO}$ A (*)
Lane 4: MES-GSC82 $ITGA6^{LO}$ B (*)
Lane 5: MES-GSC88 $ITGA6^{HI}$ A
Lane 6: MES-GSC88 $ITGA6^{HI}$ B
Lane 7: MES-GSC88 $ITGA6^{Lo}$ A
Lane 8: MES-GSC88 $ITGA6^{Lo}$ B
Lane 9: MES-GSC90 $ITGA6^{HI}$ A
Lane 10: MES-GSC90 $ITGA6^{HI}$ B
Lane 11: MES-GSC90 $ITGA6^{Lo}$ A
Lane 12: MES-GSC90 $ITGA6^{Lo}$ B
(*) Lanes displayed in the figure panel

Integrin a6 antibody chemiluminescent signal and relative membrane (exposure time 8 min) relative to membrane section #1

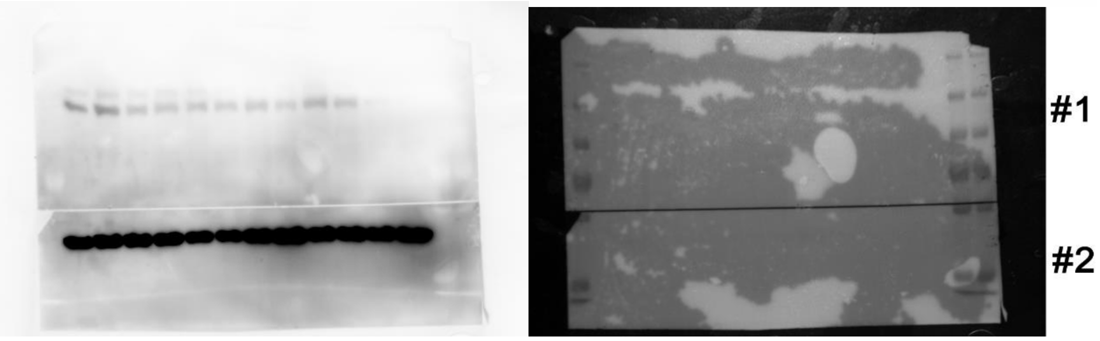

Actin antibody chemiluminescent signal and relative membrane (exposure time 1 min) relative to membrane section #2

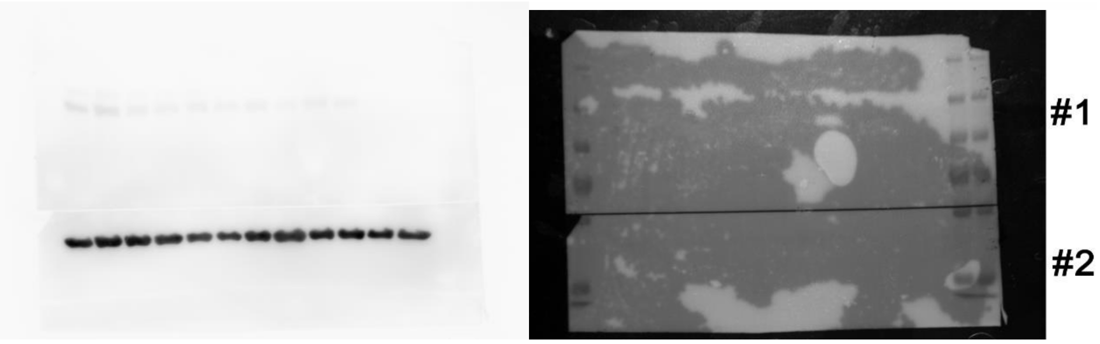

Figure S2 panel F – MES-GSC90 sorted GSC

Lane 1: MES-GSC82 ITGA6^{HI} A
Lane 2: MES-GSC82 ITGA6^{HI} B
Lane 3: MES-GSC82 ITGA6^{LO} A
Lane 4: MES-GSC82 ITGA6^{LO} B
Lane 5: MES-GSC88 ITGA6^{HI} A
Lane 6: MES-GSC88 ITGA6^{HI} B
Lane 7: MES-GSC88 ITGA6^{Lo} A
Lane 8: MES-GSC88 ITGA6^{Lo} B
Lane 9: MES-GSC90 ITGA6^{HI} A (*)
Lane 10: MES-GSC90 ITGA6^{HI} B (*)
Lane 11: MES-GSC90 ITGA6^{Lo} A (*)
Lane 12: MES-GSC90 ITGA6^{Lo} B (*)
(*) Lanes displayed in the figure panel

Integrin a6 antibody chemiluminescent signal and relative membrane (exposure time 8 min) relative to membrane section #1

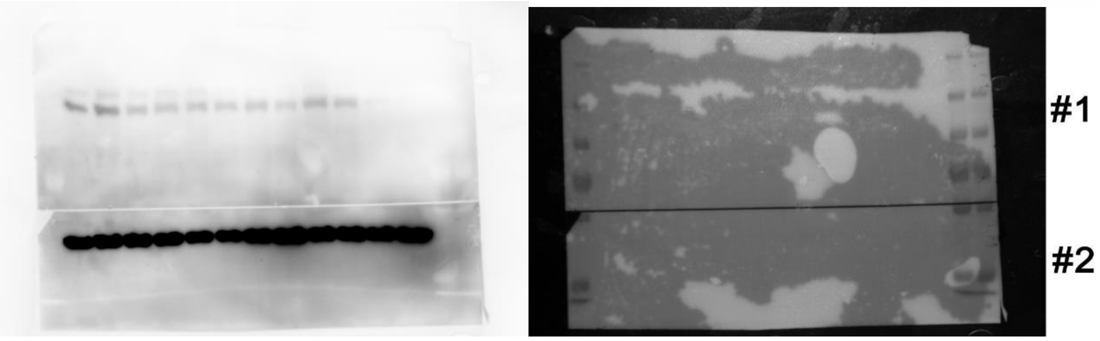

Actin antibody chemiluminescent signal and relative membrane (exposure time 1 min) relative to membrane section #2

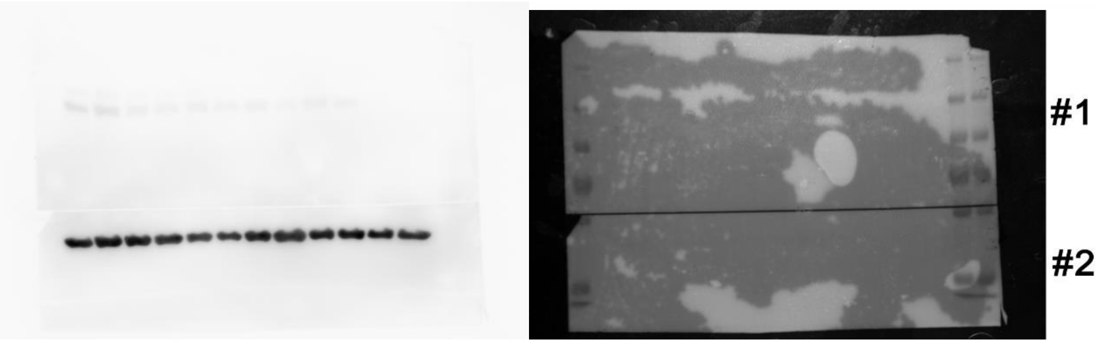

Figure 2 panel A – PN-GIC2 lentiviral *ITGA6* inhibition

Lane 1: PN-GIC2 shCTRL mix 1 A
Lane 2: PN-GIC2 shCTRL mix 1 B
Lane 3: PN-GIC2 shCTRL mix 1 C
Lane 4: PN-GIC2 shITGA6 mix 1 A
Lane 5: PN-GIC2 shITGA6 mix 1 B
Lane 6: PN-GIC2 shITGA6 mix 1 C
Lane 7: PN-GIC2 shCTRL mix 2 A (*)
Lane 8: PN-GIC2 shCTRL mix 2 B (*)
Lane 9: PN-GIC2 shCTRL mix 2 C (*)
Lane 10: PN-GIC2 shITGA6 mix 2 A (*)
Lane 11: PN-GIC2 shITGA6 mix 2 B (*)
Lane 12: PN-GIC2 shITGA6 mix 2 C (*)
(*) Lanes displayed in the figure panel

Integrin a6 antibody chemiluminescent signal and relative membrane (exposure time 7.5 min) relative to membrane section #1

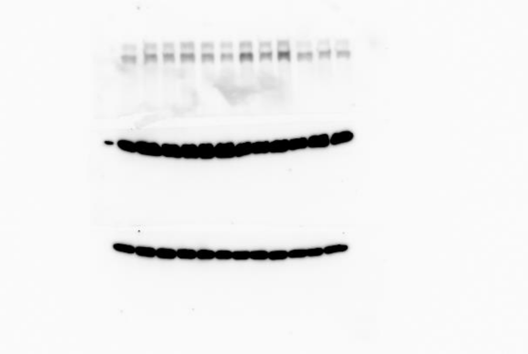

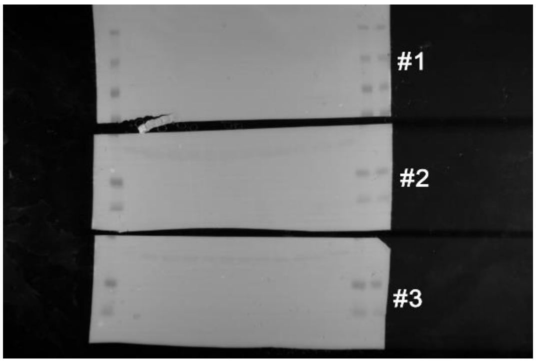

Actin antibody chemiluminescent signal and relative membrane (exposure time 1.5 min) relative to membrane section #2

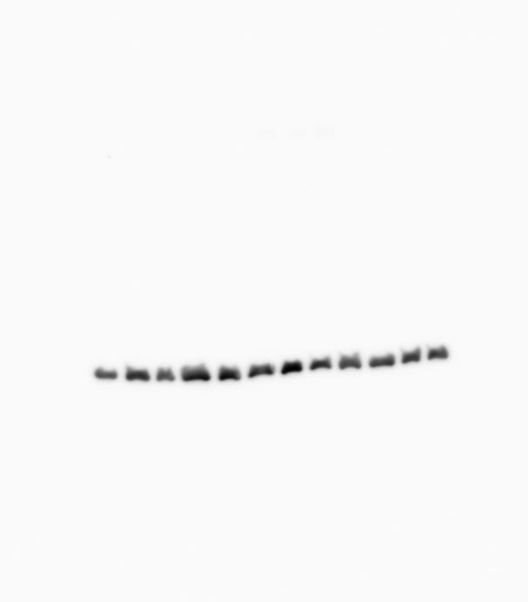

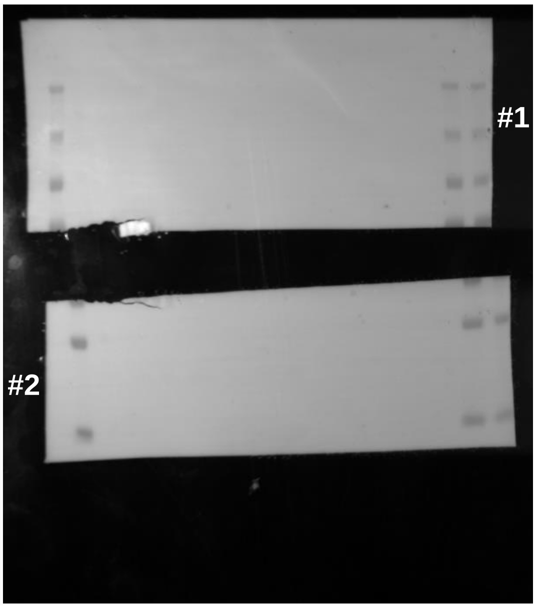

Figure 2 panel C – MES-GSC82 lentiviral *ITGA6* inhibition

Lane 1: MES-GSC82 shCTRL A (*)
Lane 2: MES-GSC82 shCTRL B (*)
Lane 3: MES-GSC82 shCTRL C (*)
Lane 4: MES-GSC82 shITGA6 A (*)
Lane 5: MES-GSC82 shITGA6 B (*)
Lane 6: MES-GSC82 shITGA6 C (*)
(*) Lanes displayed in the figure panel

Integrin a6 antibody chemiluminescent signal and relative membrane (exposure time 7 min) relative to membrane section #1

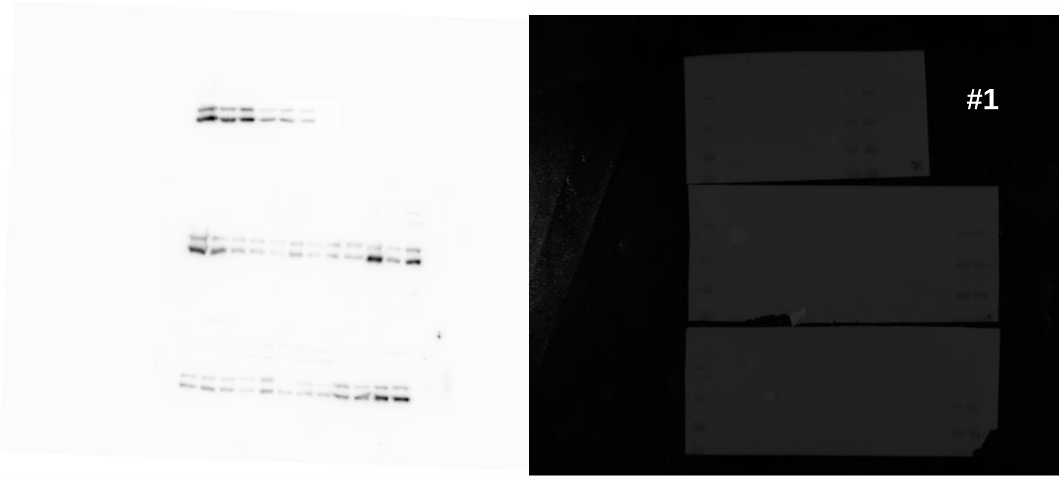

Actin antibody chemiluminescent signal and relative membrane (exposure time 40 sec)

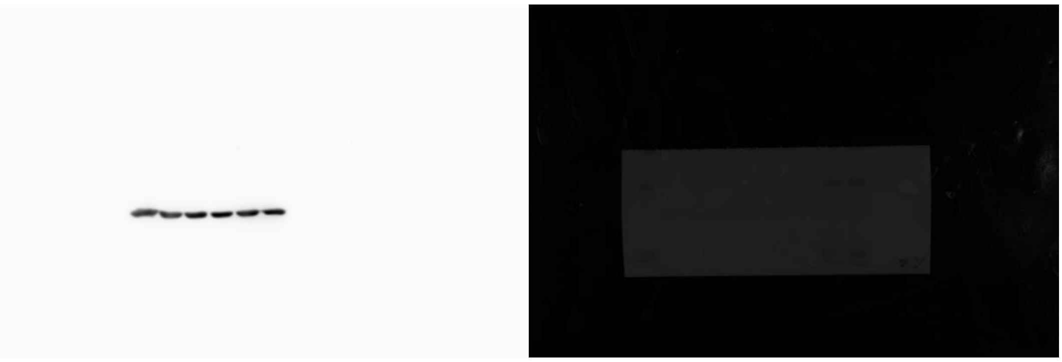

Figure 2 panel C – MES-GSC88 lentiviral *ITGA6* inhibition

Lane 1: MES-GSC88 shCTRL A (*)
Lane 2: MES-GSC88 shCTRL B (*)
Lane 3: MES-GSC88 shCTRL C (*)
Lane 4: MES-GSC88 shCTRL D (*)
Lane 5: MES-GSC88 shITGA6 A (*)
Lane 6: MES-GSC88 shITGA6 B (*)
Lane 7: MES-GSC88 shITGA6 C (*)
Lane 8: MES-GSC88 shITGA6 D (*)
(*) Lanes displayed in the figure panel

Integrin a6 antibody chemiluminescent signal and relative membrane (exposure time 9 min) relative to membrane section #1

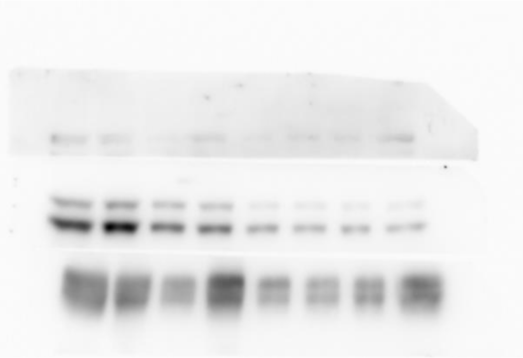

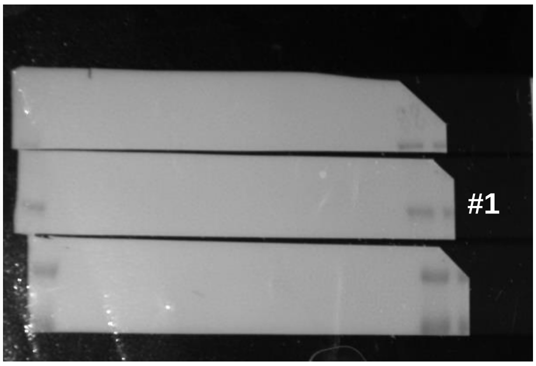

Actin antibody chemiluminescent signal and relative membrane (exposure time 4 min) relative to membrane section #1

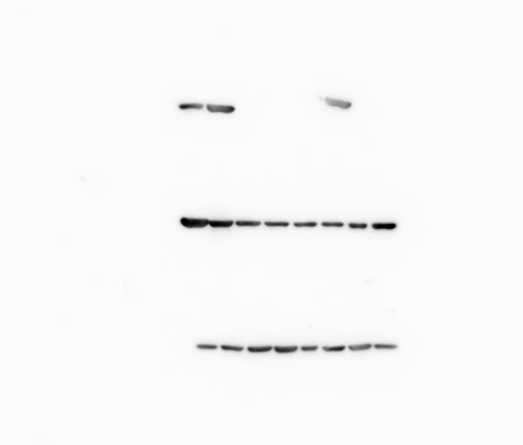

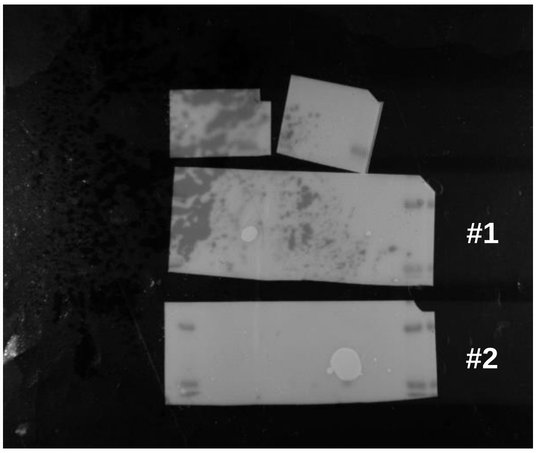

Figure 2 panel C – MES-GSC90 lentiviral *ITGA6* inhibition

Lane 1: MES-GSC90 shCTRL A
Lane 2: MES-GSC90 shCTRL B (*)
Lane 3: MES-GSC90 shCTRL C (*)
Lane 4: MES-GSC90 shCTRL D (*)
Lane 5: MES-GSC90 shITGA6 A (*)
Lane 6: MES-GSC90 shITGA6 B (*)
Lane 7: MES-GSC90 shITGA6 C (*)
Lane 8: MES-GSC90 shITGA6 D
(*) Lanes displayed in the figure panel

Integrin a6 antibody chemiluminescent signal and relative membrane (exposure time 7.5 min) relative to membrane section #1

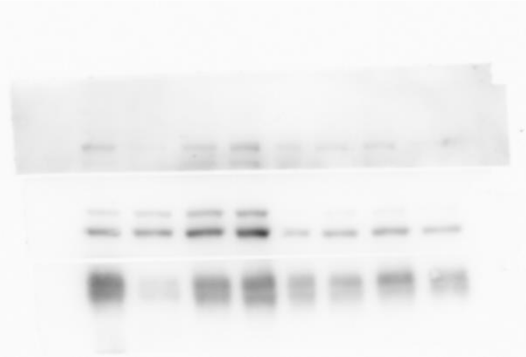

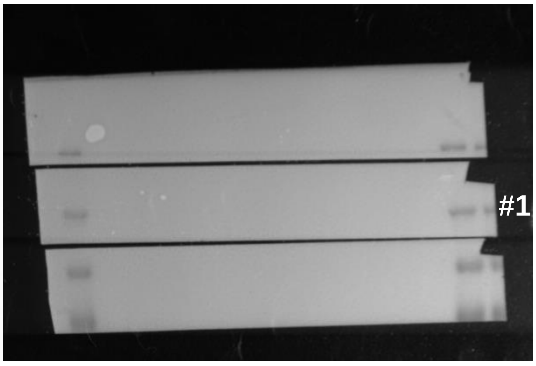

Actin antibody chemiluminescent signal and relative membrane (exposure time 4 min) relative to membrane section #2

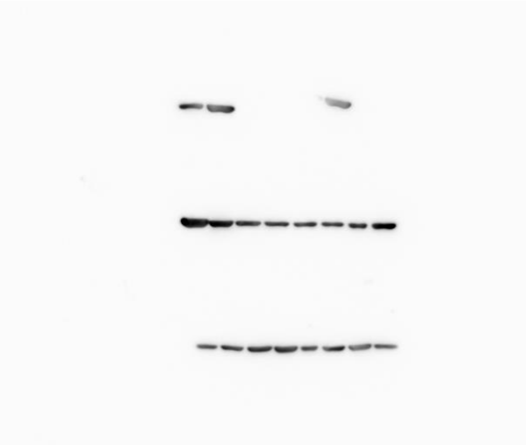

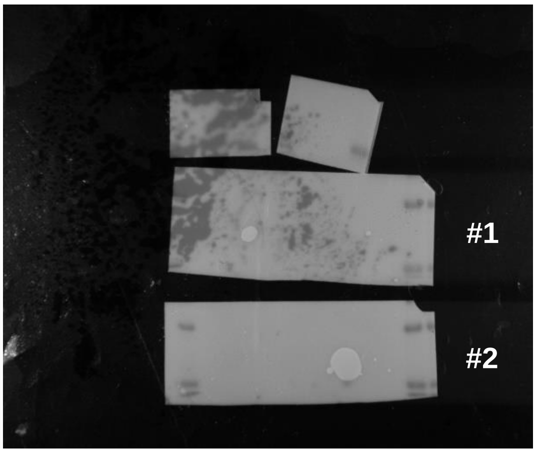

Figure S5 panel D – MES-GSC88

Lane 1: MES-GSC88 shCTRL CT A (*)
Lane 2: MES-GSC88 shCTRL 8 Gy A (*)
Lane 3: MES-GSC88 shITGA6 CT A (*)
Lane 4: MES-GSC88 shITGA6 8 Gy A (*)
Lane 5: MES-GSC88 shCTRL CT B
Lane 6: MES-GSC88 shCTRL 8 Gy B
Lane 7: MES-GSC88 shITGA6 CT B
Lane 8: MES-GSC88 shITGA6 8 Gy B
Lane 9: MES-GSC88 shCTRL CT C
Lane 10: MES-GSC88 shCTRL 8 Gy C
Lane 11: MES-GSC88 shITGA6 CT C
Lane 12: MES-GSC88 shITGA6 8 Gy C
(*) Lanes displayed in the figure panel

Integrin a6 antibody chemiluminescent signal and relative membrane (exposure time 30 seconds) relative to membrane section #1

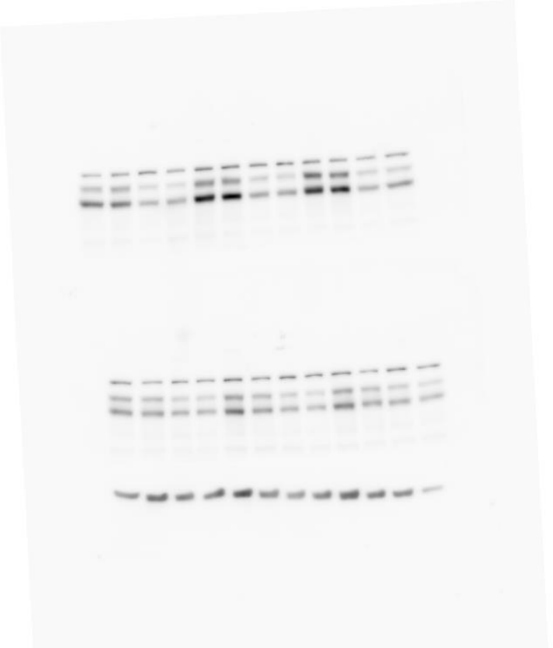

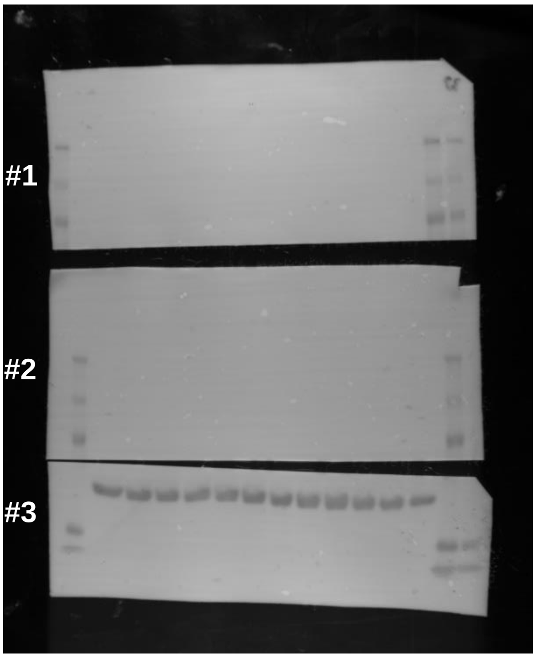

P-ERK1/2 antibody chemiluminescent signal and relative membrane (exposure time 4 min) relative to membrane section #2

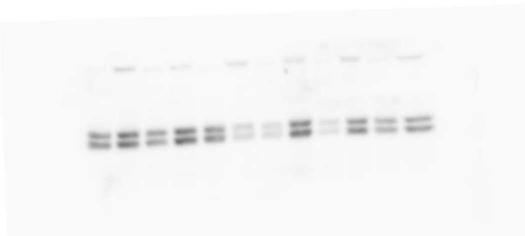

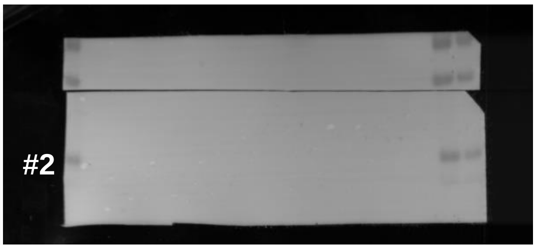

P-AKT antibody chemiluminescent signal and relative membrane (exposure time 1.5 min) relative to membrane section #2

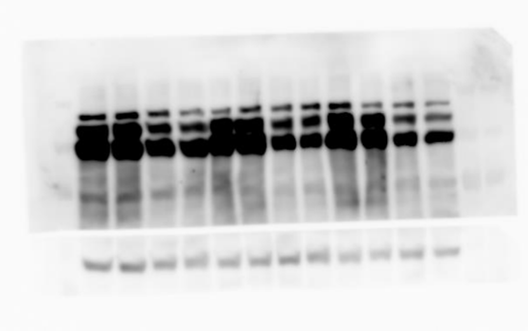

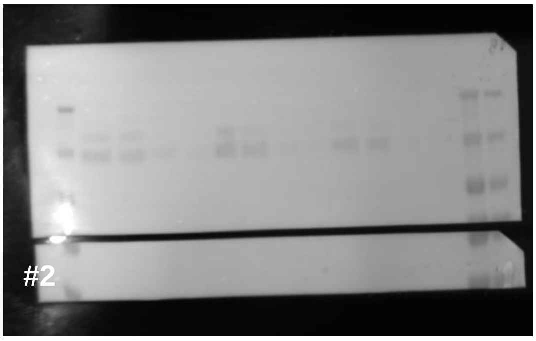

Actin antibody chemiluminescent signal and relative membrane (exposure time 30 seconds) relative to membrane section #3

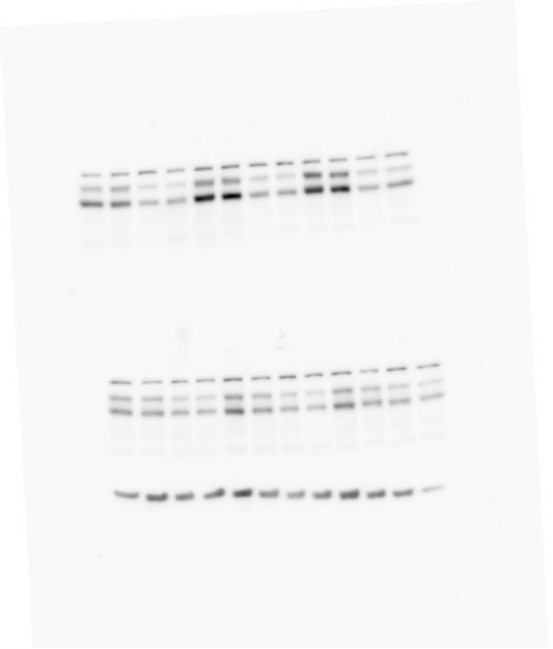

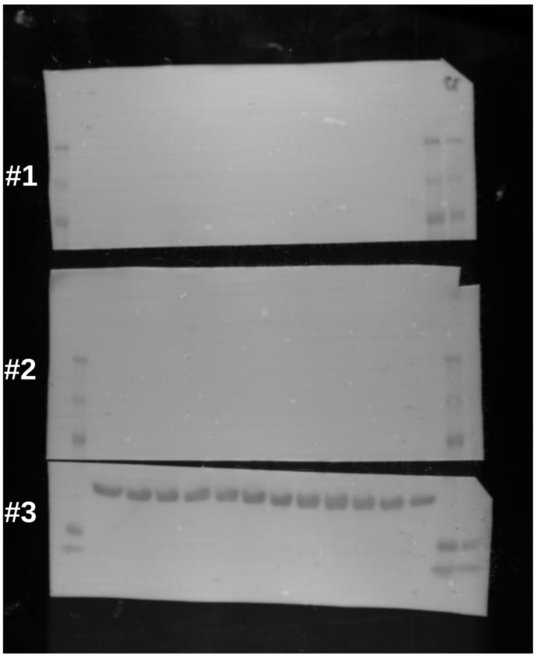

Figure S5 panel D – MES-GSC90

Lane 1: MES-GSC90 shCTRL CT A
Lane 2: MES-GSC90 shCTRL 8 Gy A
Lane 3: MES-GSC90 shITGA6 CT A
Lane 4: MES-GSC90 shITGA6 8 Gy A
Lane 5: MES-GSC90 shCTRL CT B
Lane 6: MES-GSC90 shCTRL 8 Gy B
Lane 7: MES-GSC90 shITGA6 CT B
Lane 8: MES-GSC90 shITGA6 8 Gy B
Lane 9: MES-GSC90 shCTRL CT C (*)
Lane 10: MES-GSC90 shCTRL 8 Gy C (*)
Lane 11: MES-GSC90 shITGA6 CT C (*)
Lane 12: MES-GSC90 shITGA6 8 Gy C (*)
(*) Lanes displayed in the figure panel

Integrin a6 antibody chemiluminescent signal and relative membrane (exposure time 30 seconds) relative to membrane section #2

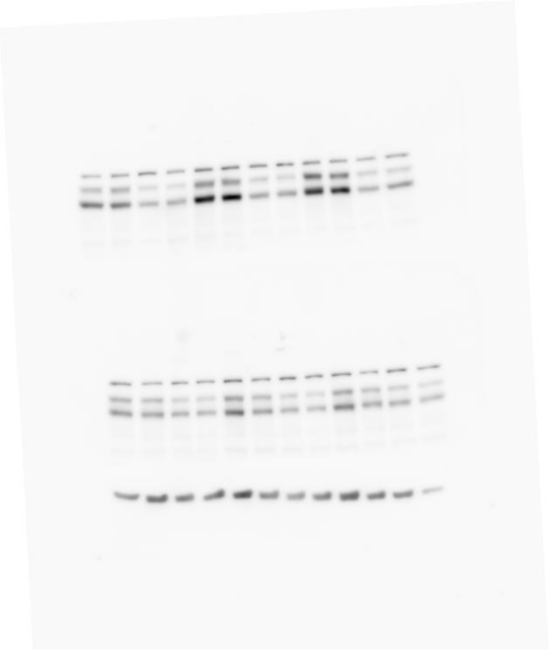

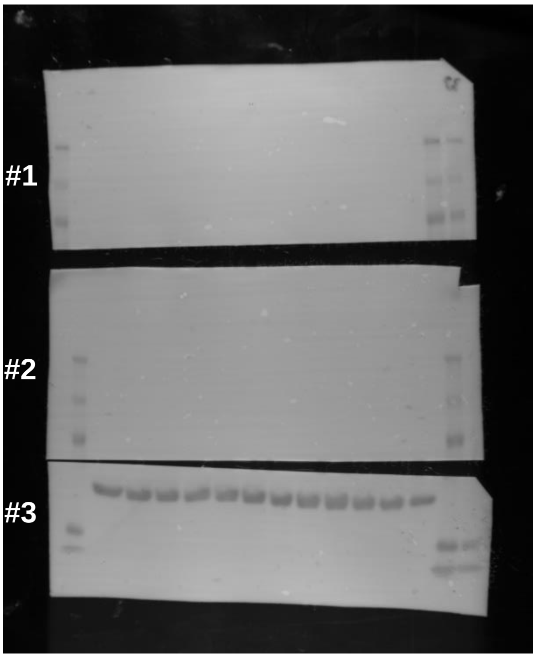

P-ERK1/2 antibody chemiluminescent signal and relative membrane (exposure time 4 min) relative to membrane section #2

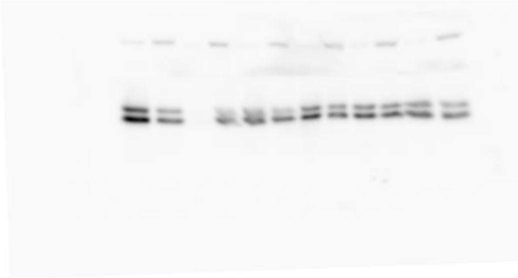

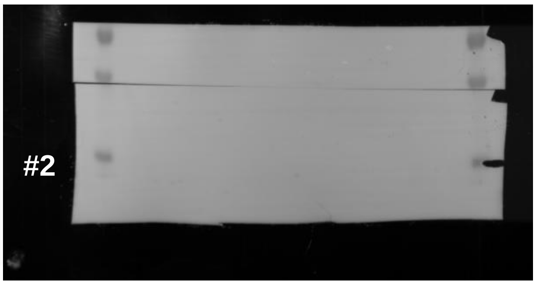

P-AKT antibody chemiluminescent signal and relative membrane (exposure time 1.5 min) relative to membrane section #2

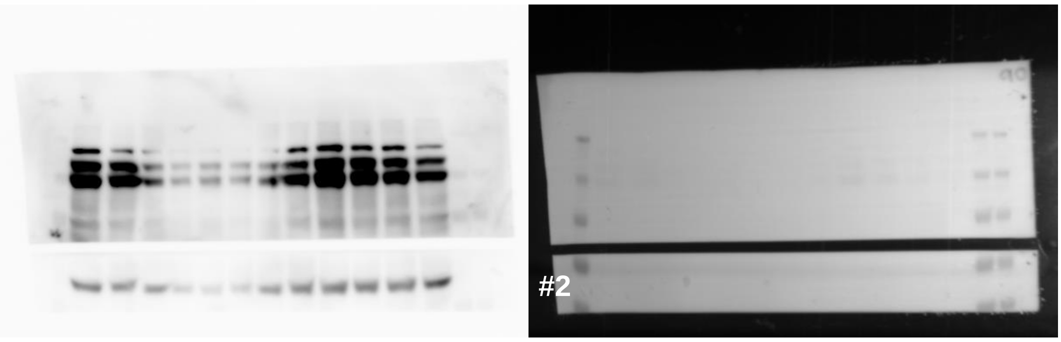

Actin antibody chemiluminescent signal and relative membrane (exposure time 30 seconds) relative to membrane section #2

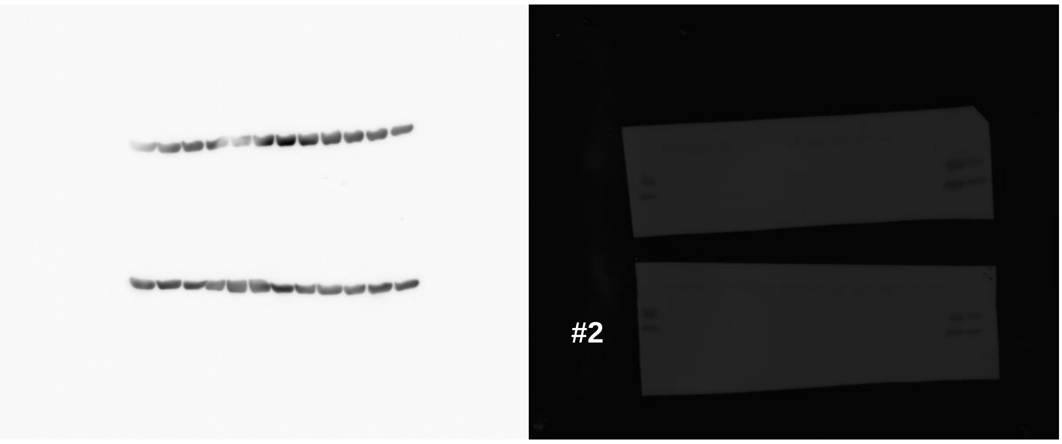

Supplement: Supplementary file 1 [file cancers-13-03055-s001.zip › cancers-1209350-supplementary/revised supp+WB/cancers-1209350 WB.pdf]
